# Electrifying Energy and Chemical Transformations with Single-Atom Alloy Nanoparticle Catalysts

**DOI:** 10.1021/acscatal.4c00365

**Published:** 2024-04-08

**Authors:** Qiang Gao, Xue Han, Yuanqi Liu, Huiyuan Zhu

**Affiliations:** † Department of Chemistry, University of Virginia, Charlottesville, Virginia 22904, United States; ‡ Department of Chemical Engineering, Virginia Polytechnic Institute and State University, Blacksburg, Virginia 24061, United States; § Department of Chemical Engineering, University of Virginia, Charlottesville, Virginia 22904, United States

**Keywords:** single-atom alloys, electrocatalysis, fuel cell reactions, water-splitting, carbon dioxide reduction, nitrate reduction

## Abstract

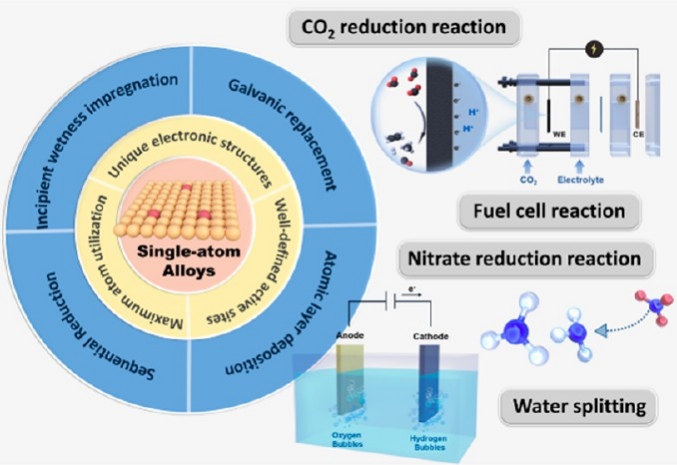

Single-atom alloys (SAAs) have attracted considerable attention as promising electrocatalysts in reactions central to energy conversion and chemical transformation. In contrast to monometallic nanocrystals and metal alloys, SAAs possess unique and intriguing physicochemical properties, positioning them as ideal model systems for studying structure–property relationships. However, the field is still in its early stages. In this Perspective, we first review and summarize rational synthesis methods and advanced characterization techniques for SAA nanoparticle catalysts. We then emphasize the extensive applications of SAAs in a range of electrocatalytic reactions, including fuel cell reactions, water splitting, and carbon dioxide and nitrate reductions. Finally, we provide insights into existing challenges and prospects associated with the controlled synthesis, characterization, and design of SAA catalysts.

## 1 Introduction

With the escalating global energy demand and growing environmental concerns, the urgency for sustainable energy and chemical transformation has grown rapidly.^1−3^ In response to the foreseen energy and chemical crisis, electrocatalytic schemes play a vital role in replacing conventional fossil-fuel-dependent routes.^4,5^ In recent years, significant advancements have been achieved in electrocatalytic fuel cell reactions,^1,4,5^ water splitting,^3,6^ carbon dioxide reduction reaction (CO_2_RR),^7−10^ and nitrate reduction reaction (NO_3_RR),^11,12^ offering promising sustainable alternatives for clean energy conversion and chemical transformation.^13,14^ Currently, nanoparticle electrocatalysts, particularly those based on precious metals, continue to be extensively utilized in these reactions. Nevertheless, their broad application faces challenges due to the high cost and limited reserves of precious metals.^15^ Consequently, it is imperative to advance the development of catalysts that not only exhibit high efficiency but, more importantly, are cost-effective to enable large-scale applications of electrochemical schemes.

Single-atom catalysts (SACs), characterized by well-defined, atomically dispersed metal atoms serving as active centers anchored onto solid supports such as oxides and carbon materials, have gained increasing attention in many important industrial reactions.^16−18^ Because of the atomic dispersion of metal atoms, SACs are deemed to be able to embrace a high atom utilization efficiency. Nevertheless, there still exist challenges in controlling the loading of single atoms in SACs and mitigating their potential aggregation under high-temperature reactive conditions.^19,20^ Recently, single-atom alloys (SAAs) have emerged as a new class of SACs, in which metal atoms, usually precious-metal-based, are atomically dispersed and alloyed within another metal host.^21,22^ Owing to the metallic interactions and atomic dispersion, SAAs showcase a unique combination of properties from both alloys and SACs. Alloys often face limitations imposed by mean-field behavior that leads to scaling relations,^23^ while conventional SACs are constrained by low metal-atom loading and atom aggregation due to the Gibbs–Thomson effect.^19,20^ SAAs featuring atomically dispersed metal atoms within a host metal, offer thermodynamic stabilization to single atoms and provide tunable electronic properties to boost catalytic performance.^21,22,24^ The well-defined active sites at the atomic level, coupled with unique electronic structures and the potential for ultrahigh atom utilization in SAAs, contribute to exceptional electrocatalytic performance and, in turn, lead to valuable insights into structure–property relationships.^25,26^

**Figure 1:**
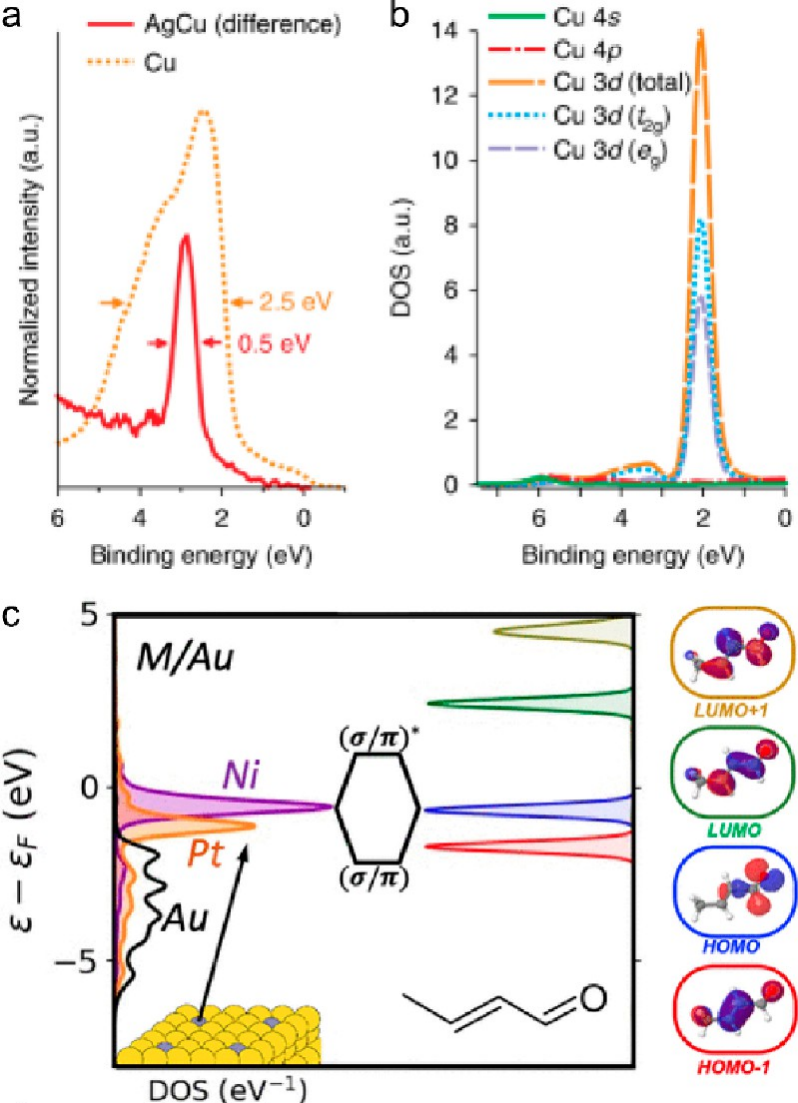
(a) The measured valence photoemission spectra of an AgCu SAA that contained 0.3 at. % Cu and a Cu reference. (b) Calculated Cu-based partial DOS of Ag_31_Cu_1_ SAA. Reproduced with permission from ref (23). Copyright 2018 Springer Nature. (c) Left: d-projected DOS for SAAs with a Au (111) host. The colored atom of the inset is the substituted site, for which d-pDOS is plotted: Ni (purple), Pt (orange), and unsubstituted Au (black). Right: Frontier and adjacent orbitals for crotonaldehyde. Reproduced with permission from ref (30). Copyright 2021 American Chemical Society.

Introducing atomically dispersed dopants into a host tailors the electronic structure of metal active sites, dictating their interactions with adsorbates in catalytic reactions.^24,27^ For example, SAAs can provide bifunctional sites for hydrogen (H_2_) dissociation at the single atom site and H spillover to the inert host metal during the hydrogenation of the C≡C triple bond.^28,29^ In addition, since SAA is within the limit of diluting alloy, the appropriate combination between single atoms and the host metal could exhibit weak wave function mixing, thus leading to an unusual and unique free-atom-like electronic state.^23,30^ The interaction of this free-atom-like state with adsorbates resembles the bonding in molecular complexes, suggesting that SAAs could bridge the gap between homogeneous and heterogeneous catalysts. A study conducted by Greiner et al. showcased the observation of free-atom-like narrow d states of Cu single atoms within the AgCu alloy using valence photoemission spectroscopy.^23^ This study revealed that the Cu 3d states in AgCu were merely one-fifth the width of those observed in bulk Cu (Figure 1a). Additionally, calculated partial density of states (DOS) suggested the near degeneracy of Cu 3d states in AgCu (Figure 1b), which, in contrast, typically experience splitting in the octahedral field of bulk Cu crystals. The authors further screened numerous alternative solutes within a Ag matrix to identify similar SAAs. Among these alloys, only four solutes (Ni, Pd, Mn, and Cr) were discovered to exhibit exceptionally narrow d bands. Likewise, the Pt and Ni single atoms exhibited narrow d-projected DOS when alloyed into the Au host (Figure 1c), which could enhance interactions with specific adsorbate frontier orbitals.^30^

The aforementioned intriguing properties such as providing bifunctional sites and narrow d-states endow SAAs with tunable adsorption energy of reaction species, and the potential to break linear scaling relations, ultimately leading to enhanced electrocatalytic performance.^23,30^ Moreover, the formation of metallic bonds between the doping atom and the host metal matrix contributes to the improved stability of the single atom within SAA, making it less susceptible to phase separation compared to other types of SACs.^31,32^

This Perspective aims to highlight recent advancements in the design, synthesis, characterization, and application of SAA nanoparticle catalysts for diverse electrochemical reactions. It begins by reviewing and discussing the synthesis and characterization techniques for SAAs. We then cover and summarize the latest developments in utilizing SAA nanoparticle catalysts across various electrochemical reactions, including oxygen reduction reaction (ORR), alcohol/formic acid oxidation reaction, oxygen evolution reaction (OER), hydrogen evolution reaction (HER), CO_2_RR, and NO_3_RR. Finally, the challenges and perspectives for the future development of SAAs in energy/chemical-conversion electrocatalysis are presented.

## 2 Chemical Synthesis of SAA Nanoparticles

Thermodynamically, the SAA formation can be driven by the difference in surface free energy between the single-atom metal and host metal, negative mixing enthalpy, and the relief of strain upon alloying.^33^ In situations where the formation of SAAs is kinetically limited at low temperatures, various metastable surface structures can be captured.^33^ The crucial factor for SAA formation lies in ensuring that the interaction between single atoms and the host is sufficiently robust to maintain isolated and stable single sites.^34,35^ Furthermore, a positive aggregation energy for a cluster relative to its SAA signifies the stability of isolated dopant metals when compared to segregated dopant domains.^36^ Efficient and straightforward approaches for synthesizing SAA catalysts are essential. Therefore, a comprehensive understanding of the synthesis process using various methods is of paramount significance. Generally, the synthesis strategies of alloy nanoparticles can be extended to SAA nanoparticles. Metal atoms tend to disperse homogeneously in the host metal nanoparticles when they share similar sizes and bond-formation energies. By reducing the loading amount or concentration of the single-atom component, SAA nanoparticles can be synthesized. In this section, we summarize several synthesis techniques for SAAs, including incipient wetness impregnation, galvanic replacement, sequential reduction, and atomic layer deposition. This Perspective does not cover the synthesis methods conducted under ultrahigh vacuum conditions, as they have been thoroughly reviewed elsewhere.^22,37^

### 2.1 Incipient Wetness Impregnation

Incipient wetness impregnation is a widely utilized two-step process for the synthesis of monometallic and alloy nanoparticles, and it has recently been extended to the SAA nanoparticle synthesis. This method has gained significant attention due to its simplicity and effectiveness.^22^ Typically, the incipient wetness impregnation process begins with the preparation or acquisition of a porous solid support material with a high surface area, such as silica gels,^38−41^ metal oxides,^42−44^ and carbides.^45^ The solid support is then impregnated with a precursor solution containing the desired metal composition. Following impregnation, the material is subjected to drying and other subsequent treatments, such as calcination or reduction. The key to a successful synthesis of SAA nanoparticles using incipient wetness impregnation is the precise control of the precursor amount and reaction temperature.

**Figure 2:**
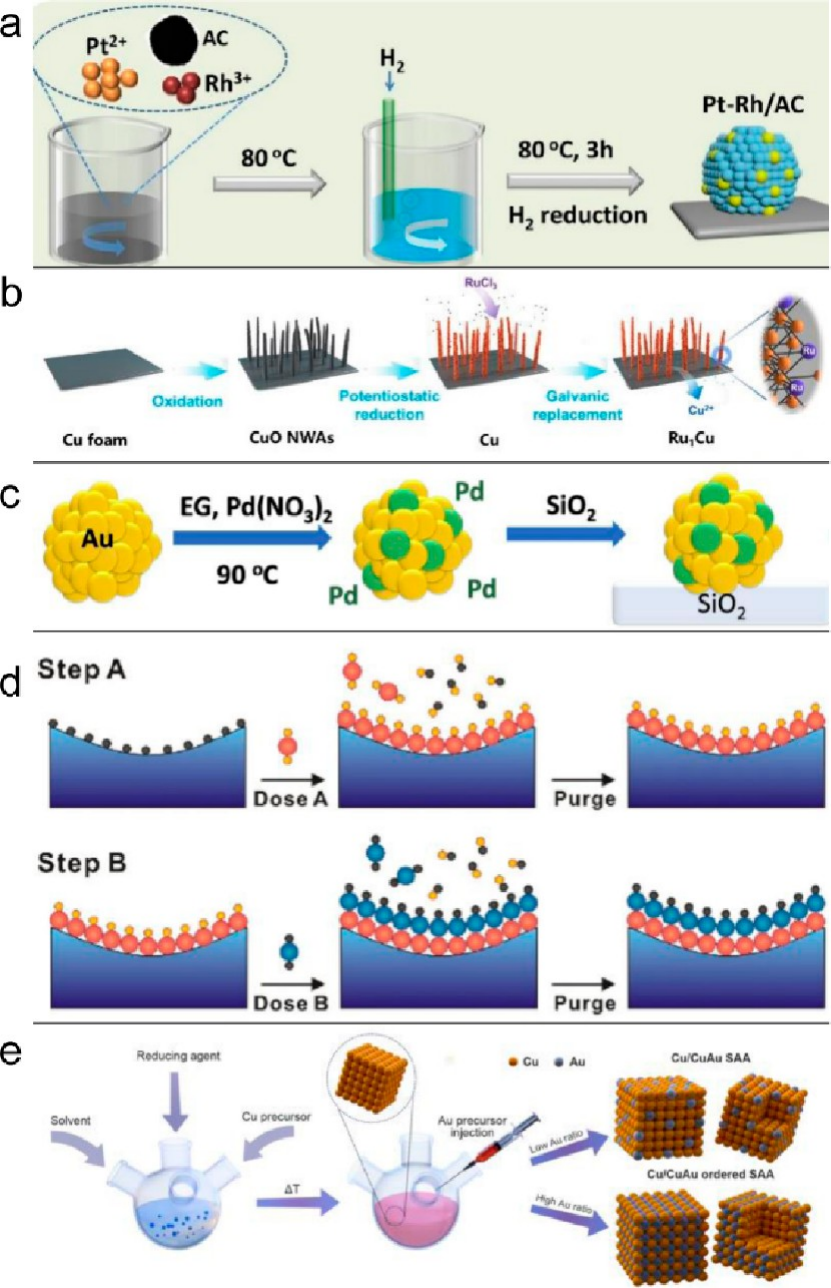
Schematic illustrations of SAA synthetic strategies. (a) Fabrication of Pt–Rh/AC via incipient wetness impregnation. Reproduced with permission from ref (45). Copyright 2023 Elsevier. (b) Synthetic route for the RuCu SAA by galvanic replacement. Reproduced with permission from ref (52). Copyright 2022 Wiley. (c) PdAu/SiO_2_ SAA synthesis via sequential reduction. Reproduced with permission from ref (55). Copyright 2021 Springe Nature. (d) The atomic layer deposition process. Reproduced with permission from ref (58). Copyright 2022 American Chemical Society. (e) The core/shell Cu/CuAu SAA nanocrystal synthesis. Reproduced with permission from ref (65). Copyright 2023 Springer Nature.

Lu et al. employed the incipient wetness impregnation method to prepare SAA Pt–Rh supported on activated carbon.^45^ The activated carbon was dispersed in deionized water, and Pt and Rh precursors were added at an elevated temperature under a reducing atmosphere. The Pt^4+^ and Rh^4+^ were then simultaneously reduced by H_2_ and formed Pt–Rh SAA nanoparticles (1 wt % Pt and 0.05 wt % Rh) on the activated carbon (Figure 2a). Additionally, Zhang et al. synthesized Pt–Cu/SiO_2_ SAA nanoparticles using the incipient wetness impregnation method.^38^ By carefully controlling the introduction of different amounts of the Pt precursor (Pt(NH_3_)_4_(OH)_2_) into Cu/SiO_2_ during the wet impregnation, they achieved various weight percentages of Pt, ranging from 0.06% to 3%. While wetness impregnation offers a one-step, straightforward process for synthesizing SAAs, the control of dopant metal loading needs to be meticulously managed at a low level to prevent the agglomeration of single atoms. This limitation can impact the dispersion density of single atoms on the host metal. Moreover, the possibility of the migration of dopant metal from the host metal to the support is a potential issue that has not been thoroughly explored or discussed in prior studies.

### 2.2 Galvanic Replacement

Galvanic replacement is a widely adopted chemical and electrochemical synthesis method for nanostructures in which one metal element is replaced by another active metal element in the solution.^46^ This process is driven by the spontaneous redox reaction between two metal redox pairs with a difference in the standard reduction potentials. This reaction leads to the displacement of metal atom sites on the nanostructure, with the metal component from the solution having a higher reduction potential and replacing those with a lower reduction potential. Subsequently, the surface metal atoms become oxidized to metal ions, which then dissolve into the solution. Meanwhile, the metal ions from the solution undergo reduction, with most cases involving their deposition onto the surface of the nanostructures. This deposition occurs due to a lower energy barrier of heterogeneous nucleation compared to that of homogeneous nucleation.^22^ The reaction rate is typically governed by the degree of difference in the reduction potentials. The larger the difference, the faster the displacement.^47^ The morphology of deposited metals and the composition of the final structure are dictated by multiple factors, including the reaction temperature, precursor ratio, capping agents, and whether or not an additional reducing agent has been used. The size of the substrate structure could also cause a drastic change in the thermodynamics.^46−49^ Note that when a reducing agent is present in the solution, co-reduction and galvanic replacement may coexist and compete. This might lead to the formation of self-nucleated metal clusters/particles or redeposition of the dissolved metal ions onto the substrate structure.^50,51^ By a judicious choice of the metal substrate and the metal precursor in the solution, along with precise control of the ratio and reaction conditions, galvanic replacement can be harnessed to alloy single atoms into nanostructures.

Duan et al. synthesized RuCu SAA catalysts using a facile chemical oxidation method followed by electrochemical reduction and galvanic replacement, as shown in Figure 2b.^52^ The pristine Cu foam was first oxidized and transformed into CuO nanowire arrays (NWAs) under alkaline conditions. Subsequently, through potentiostatic reduction, oxygen was removed from the catalyst, forming Cu NWAs. Finally, the Cu NWAs were immersed in a RuCl_3_ solution to enable the galvanic replacement process and yield RuCu SAA catalysts. The key to achieving Ru single atom dispersion on the NWAs surface is an extremely low Ru concentration and a short replacement reaction duration. Moreover, Wei et al. successfully synthesized RuNi SAA on amorphous Al_2_O_3_ substrates, achieving uniform dispersion of Ru atoms on the Ni surface through the galvanic replacement method, with minimal changes in morphology and surface area.^53^ Galvanic replacement has gained considerable prominence as a method for synthesizing surface alloys, primarily due to its process simplicity. This method has already enabled the synthesis of multimetallic, and now SAA nanostructures in a single step with remarkably short reaction periods. Unlike incipient wet impregnation, galvanic replacement does not require additional reducing agents. However, the scope of SAAs synthesizable by galvanic replacement is limited to some extent due to the necessity of appropriate redox pairs.

### 2.3 Sequential Reduction

Similar to the galvanic replacement, where a nanostructure substrate is prepared first, sequential reduction in the chemical synthesis of SAA catalysts involves a consecutive series of reduction reactions performed in a specific sequence to introduce dopant metal atoms onto the surface of the host metal. In contrast to the galvanic replacement method, certain metals with lower reduction potentials may not undergo direct reduction. Instead, they might be reduced by reductive intermediates first and then grow on the host metal surface during sequential reduction. In the case of the presence of small amounts of metals such as Ni^54^ and Pd,^55−57^ sequential reduction can effectively introduce them onto the surface of host metal nanoparticles, such as Au. Wu et al. successfully dispersed single-atom Ru on PtCu_*x*_/Pt core–shell structures via acid etching and electrochemical leaching.^32^ The acid treatment transformed Ru-doped PtCu_3_ nanoisland chains into an acid-stable Ru-doped PtCu tubular core–shell structure, and the electrochemical leaching process removed excess Cu atoms to form the final Ru-Pt_3_Cu core–shell nanoisland chains. Similarly, Sykes et al. prepared the PdAu/SiO_2_ SAA by reducing Pd(NO_3_)_2_·*x*H_2_O in the presence of Au nanoparticles under N_2_ flow at 90 °C for 8 h, followed by the introduction of fumed silica into the resulting solution (Figure 2c).^55^ The precursor introduction temperature was found to significantly impact the formation of SAAs. As an example, intermetallic Pt_3_Sn nanoconcaves were initially prepared as the template and subsequently combined with Ru(acac)_3_ in the presence of N,N-dimethylformamide at a temperature of 150 °C to yield Ru-Pt_3_Sn SAA nanoconcaves.^31^ When the temperature was raised to 170 °C, the presence of small nanoparticles on the active carbon surface was observed, suggesting the self-nucleation of Ru domains.

### 2.4 Atomic Layer Deposition

Atomic layer deposition (ALD) is a chemical vapor deposition synthesis method that allows for precise control over atom-scale growth. ALD follows a cyclic process involving the exposure of vapor precursors and the purge of residual precursor molecules and byproducts (Figure 2d).^58^ In this process, the substrate is sequentially exposed to different precursor gases, and each exposure undergoes a self-limiting reaction. The self-limiting nature of ALD is determined by the available surface reaction, resulting in the deposition of only one atomic layer before the reaction ceases. During the ALD, the precursor is introduced by a carrier gas, such as H_2_,^59^ N_2_,^60^ or O_2_,^61^ and exposed to the substrate, resulting in the formation of a single atom layer. The excess unreacted precursor and byproducts are then purged by an inert gas, e.g., N_2_ or Ar (Step A). Subsequently, another precursor pulse is introduced, followed by a purge with an inert gas to prepare for the next cycle (Step B). By repeating these steps, only one atomic layer is deposited during each cycle, leading to the formation of a precisely controlled film on the support until the desired number of layers is achieved. This unique characteristic of ALD allows for exceptional control over layer thickness and composition, facilitating the formation of conformal films on diverse substrates and enabling penetration on high-surface-area substrates.^62^ Recently, ALD has been employed in SAA synthesis. For example, Lu et al. successfully dispersed Pd atoms on Ni nanoparticles (∼3.5 nm) supported on SiO_2_ via an ALD method.^59^ The Pd atoms were selectively deposited on the surface of ∼3.5 nm Ni nanoparticles without any nucleation on SiO_2_. The coverage of Pd atoms (0.98–3.5 wt %) on the Ni nanoparticles surface was controlled by adjusting the number of ALD cycles. Similar to the incipient wetness impregnation, a primary challenge in synthesizing SAA via ALD is loading single atoms onto a large surface area support without aggregation. To prevent aggregation, the low loading of single atoms typically restricts the single atom density on the support surface, generally to less than 5%. Consequently, maintaining the stable single-atom form on the support surface and simultaneously increasing the single-atom loading pose challenges for the future synthesis of SAA via ALD.

### 2.5 Synthesis of Well-Defined Ordered SAA Nanocrystals

The methods discussed for SAA synthesis have encountered challenges related to product heterogeneity. Ensuring that all products are SAA nanoparticles without forming other impurities has been a significant challenge and is sometimes overlooked. Developing synthetic strategies for well-defined, monodisperse nanoparticles could potentially address this issue, but achieving this goal has proven to be difficult. Ordered intermetallic SAAs can be used to disrupt the continuous arrangement of metal atoms, creating a structural motif that contains a high density of individual atoms compared with conventional SAAs.^63^ Moreover, the uniform metal atom arrangement and well-defined crystal structure provide strong d–d orbital interaction, which renders these ordered SAA nanocrystals better electrocatalytic stability against chemical oxidation and etching compared to the disordered SAAs.^64^ The common approach to preparing ordered intermetallic alloys typically involves synthesizing nanoparticles with uniform size and composition through solution-phase synthesis, followed by annealing at the proper temperature to transform a disordered alloy into an ordered alloy. This process requires multiple steps and a harsh environment for the ordered SAA formation. Consequently, synthesizing ordered SAA in simple steps under mild experimental conditions remains a challenging task at present.

Recently, our group synthesized two different structures of core/shell Cu/CuAu SAA nanocubes and Cu/CuAu ordered intermetallic SAA nanocubes with controllable shell thickness, surface structure, and Au atom density (Figure 2e).^65^ With the CuAu SAA growth on the Cu nanocube surface, a 3.7 wt % Au doping led to the dilute dispersion of Au atoms into the face-centered cubic (fcc) Cu structure. In contrast, 7.5 wt % Au in Cu/CuAu led to the formation of an ordered SAA shell with the body-centered tetragonal intermetallic CuAu, in which Au atoms are completely isolated by Cu atoms. Similarly, Yu et al. designed ultrathin intermetallic SAA InPd bimetallenes with a few atomic layers. The fcc Pd metallene was synthesized first, followed by a co-reduction and phase transformation process to form the PdIn body-centered cubic (bcc) structure.^66^

## 3 Characterization of Single-Atom Alloys

Characterizing SAAs plays a crucial role in facilitating the fundamental understanding of the structure and consequent properties of SAAs. Certainly, postsynthesis, a crucial task is to prove and confirm the presence of only single atoms isolated in SAAs. Achieving structural interrogation at the atomic scale necessitates the application of advanced and multimodal microscopy and spectroscopy techniques. Microscopy can provide evidence of the atomic structure of SAA, while spectroscopy can identify the presence of dopant–host bonds. Both techniques provide invaluable information about the structural and compositional aspects of the materials and aid in understanding the catalytic performance of SAAs. Notably, aberration-corrected high-angle annular dark field scanning transmission electron microscopy (HAADF-STEM) enables the direct visualization of atom dispersion. Under sufficient “Z-contrast” between the host metal and dopant metal, it becomes possible to directly reveal the dopant atom distribution among the host metal, providing a means to verify the structure of an SAA. Meanwhile, X-ray probes, especially extended X-ray absorption fine structure (EXAFS) spectroscopy, offer insights into the coordination numbers, bond distances, and electronic states. The suggestion of an SAA structure is plausible when only dopant–host bonds and host–host bonds are present, while dopant–dopant bonds are absent.

### 3.1 HAADF-STEM

**Figure 3:**
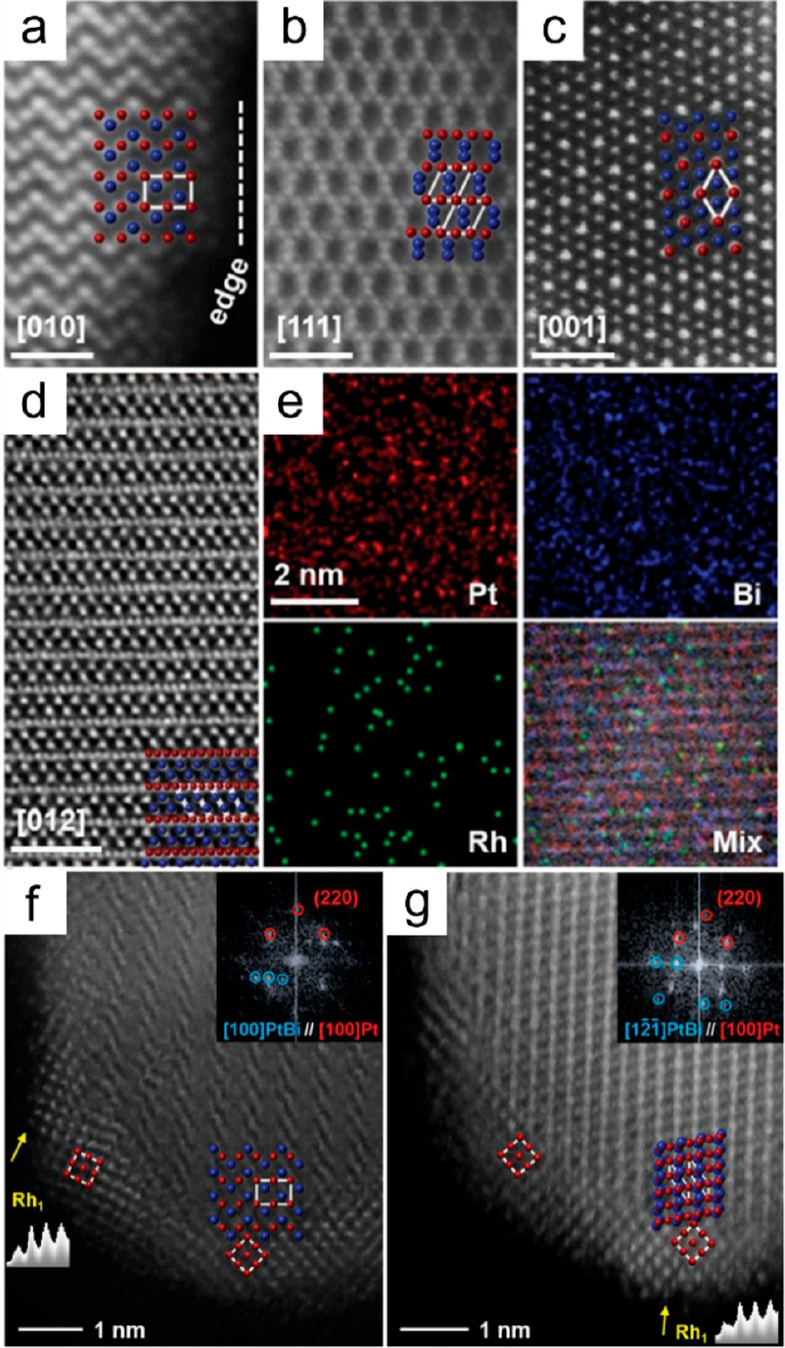
HAADF-STEM images of PtBi-Rh nanoplates and PtBi@PtRh nanoplates. (a–d) Aberration-corrected HAADF-STEM images of PtBi-3.6%Rh nanoplates. (e) High-resolution EDS mapping images for Pt, Bi, and Rh elements. (f, g) Aberration-corrected HAADF-STEM images of PtBi@PtRh nanoplates with corresponding FFT patterns and line intensity profiles analyses. Reproduced with permission from ref (71). Copyright 2021 Wiley.

Aberration-corrected STEM is an advanced microscope technology that enables the direct observation of the atomic arrangement of materials. The brief fundamental principle of STEM involves a high-energy electron beam being focused onto a small area and fired through a thin sample, collecting signals from scattered electrons and ionized atoms as the beam scans the sample, finally forming a two-dimensional map.^67^ When collecting only the electrons scattered at large angles, typically between 50 and 70 mrad, the imaging mode is referred to as HAADF-STEM.^68^ The aberration correctors enhance the signal, reducing the collection time from hours to under a minute and improving the signal-to-noise ratio to make the bonding feature visible.^69^ The signal intensity in HAADF-STEM is determined by the atomic number of the elements investigated. With the heavy atoms in the sample presenting brighter (higher Z-contrast), the distribution of elements can be revealed.^70^ For instance, Figure 3 illustrates the HAADF-STEM images of PtBi-Rh SAA nanoplates and core@shell PtBi@PtRh SAA nanoplates, where the Pt and Bi sites are distinctly visible as bright dots.^71^ Specifically, Figures 3a–d presents atomic resolution HAADF-STEM images of PtBi-Rh nanoplates viewed from [010], [111], [001], and [012] zone axes, respectively. The atomic structure of Pt_49.3_Bi_47.1_Rh_3.6_ exhibits an alternating atomic stacking arrangement consistent with the ordered intermetallic structure of PtBi, while the uniform distribution of Rh atoms within the nanoplates suggests their position along Pt or Bi atomic columns rather than in hollow sites. In addition to imaging and structural analysis, HAADF-STEM also enables elemental mapping and compositional analysis through high-resolution energy-dispersive X-ray spectroscopy (EDS), providing information about the distribution of elements within the sample. For example, in Figure 3e, EDS mapping shows uniformly distributed Rh atoms on the nanoplates. Subsequent electrochemical dealloying leads to the transformation of PtBi-Rh nanoplates into PtBi@PtRh core@shell nanoplates (Figure 3f–g). This process involves the removal of surface Bi from the PtBi unit cell, followed by the formation of fcc-Pt layers on the surface. The fcc-Pt (110) plane, with a larger average lattice spacing of 1.448 Å compared to the normal Pt (110) plane, demonstrates the presence of tensile strain in the Pt shells on PtBi nanoplates.

HAADF-STEM does face certain technical limitations. For instance, the limited field depth makes it challenging to focus on features at different depths within the sample. It is also difficult to distinguish elements with similar atomic numbers.^72^ Additionally, when a high-energy electron beam passes through samples, electron–atom and electron–electron interactions may result in direct atomic displacement and chemical bond breaking in the sample, a phenomenon known as beam damage.^73^ Moreover, HAADF-STEM can only capture information about the sample’s structure in the plane which is perpendicular to the electron beam’s path, lacking information on the sample’s internal structure in the *Z*-axis direction. Therefore, the conventional image obtained by HAADF-STEM represents a two-dimensional projection of the full three-dimensional structure of the specimen unless advanced techniques such as depth sectioning or electron tomography are applied.^74^

### 3.2 X-ray Absorption Spectroscopy

**Figure 4:**
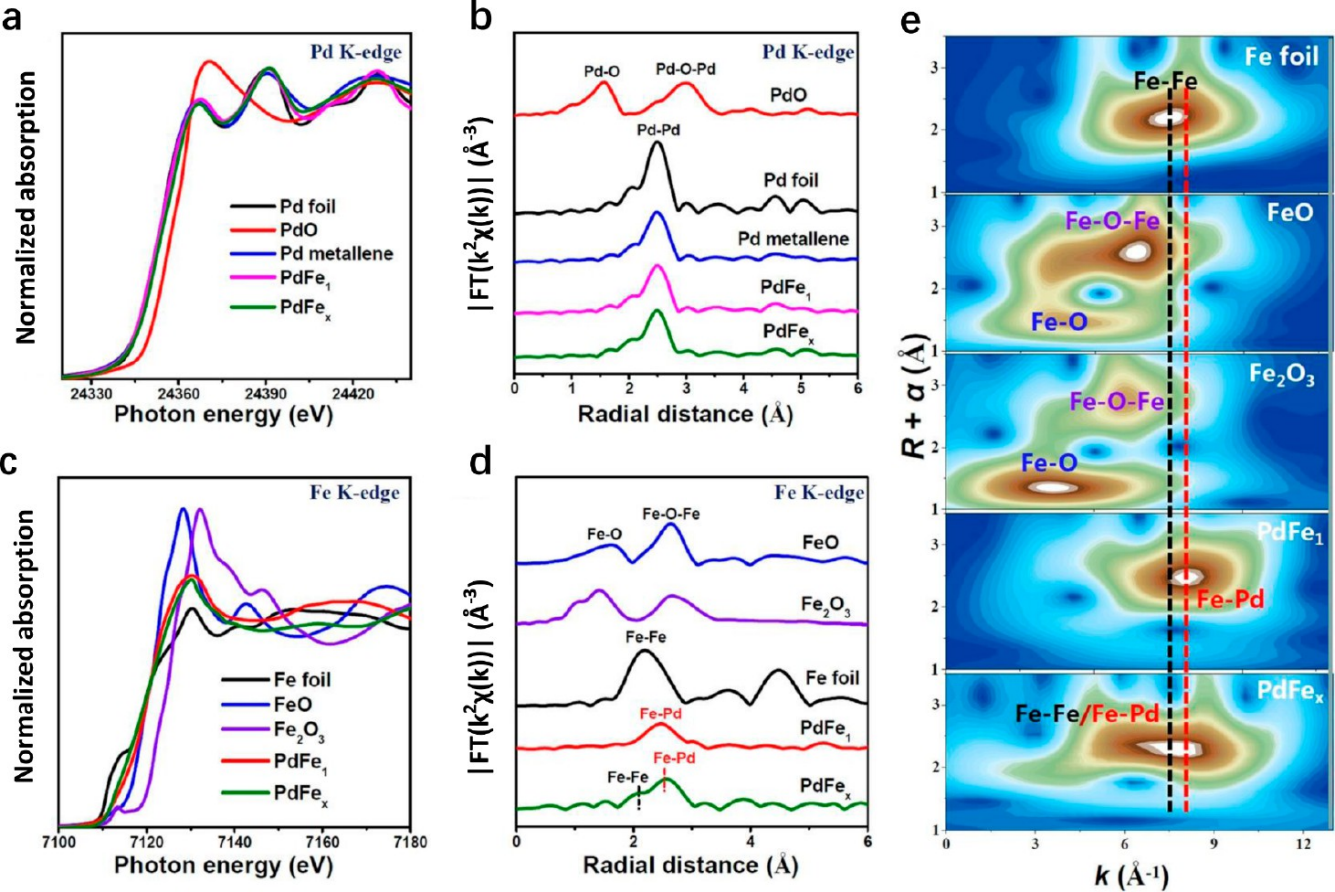
(a) Pd K-edge XANES. (b) Magnitude of the Fourier-transformed EXAFS spectra of Pd metallene, PdFe_1_, PdFe_*x*_, and reference samples of Pd foil and PdO. (c) Fe K-edge XANES. (d) Magnitude of the Fourier-transformed EXAFS spectra of PdFe_1_, PdFe_*x*_, and reference samples of Fe foil, FeO, and Fe_2_O_3_. (e) WT contour maps of PdFe_1_, PdFe_*x*_, and reference samples of Fe foil, FeO, and Fe_2_O_3_. Reproduced with permission from ref (78). Copyright 2022 Wiley.

While STEM can directly image single atoms, it is limited to providing local information for specifically selected areas. In contrast, X-ray absorption spectroscopy (XAS) offers average information about the coordination environment of the entire material. In particular, EXAFS offers detailed insights into the atomic environment of specific atoms within a sample.^75^ This includes parameters such as distances between neighboring atoms, coordination numbers, bond lengths, and bond angles.^76^ XAS, including EXAFS, enables the verification of bonds between dopants and hosts, providing direct structural information that complements other commonly used probes such as infrared spectroscopy (IR), nuclear magnetic resonance, and X-ray diffraction.^77^Figures 4a–b show the Pd X-ray absorption near-edge structure (XANES) and EXAFS of PdFe_1_ SAA, PdFe_*x*_, PdO, Pd metallene, and Pd foil structure, respectively.^78^ Compared with XANES spectra obtained from Pd foil, it is evident that the PdFe and Pd metallene absorption edges align closely with that of Pd instead of PdO (Figure 4a). Processing of the EXAFS data (Figure 4b) reveals that the Pd–Pd coordination bond experiences minimal changes in the presence of PdFe compared to the Pd metallene, indicating that the incorporation of Fe single atoms in PdFe does not significantly influence the Pd valence state or coordination structure. Figure 4c–d shows the Fe XANES and EXAFS of PdFe_1_ SAA, PdFe_*x*_, FeO, Fe_2_O_3_, and the Fe foil structure, respectively. The absorption edge of PdFe_1_, located between that of FeO and Fe foil, suggests that the valence state of Fe ranges between 0 and 2, indicating electron transfer from Fe to Pd (Figure 4c). Although the authors assert that the Fe EXAFS spectra exclusively exhibit one dominant peak corresponding to the Fe–Pd coordination bond in PdFe_1_ SAA (Figure 4d), without the presence of Fe–Fe and Fe–O coordination bonds, as reflected in the wavelet transform (WT) profiles (Figure 4e), and indicate the atomic distribution of Fe in PdFe_1_, it is important to note that assigning the peak solely to the Fe–Pd coordination bond based on peak positions in EXAFS spectra is not sufficiently rigorous to prove the existence of Fe single atoms. It is also important to clarify that peak positions in EXAFS spectra do not precisely represent the bond distances of the samples. Furthermore, in the analysis of SAA catalysts, it might be insufficient to solely demonstrate the fitting of M–N (where M is the dopant metal and N is the host metal). Better data analysis practice involves not only fitting the M–N bond but also comparing the fitting data with the addition of M–M scattering paths to achieve an optimal model with statistical evidence.^79^

In addition to the aforementioned characterization methods, IR spectroscopy using probe molecules, such as CO, can directly identify single-atom dispersions on the support. Since the vibration frequencies of probe molecules absorbed on different sites are distinct and highly dependent on the adsorption configuration, the dispersion of single atoms can be identified through the behaviors of CO adsorption/desorption.^21,26^

## 4 Electrocatalytic Performance of SAAs

SAAs exhibit promising potential as alternatives to traditional precious metal catalysts due to their ability to maximize atom utilization, possess unique electronic structures, and provide well-defined active sites. These properties are conducive to breaking the linear scaling relations and tuning the adsorption energy of reaction species and pathways.^23,30^ In this section, we discuss their electrocatalytic applications in detail. We also offer insights into the structure–activity relationship and the rational design of electrocatalysts with high activity, selectivity, and durability.

### 4.1 Fuel Cell Reactions: Oxygen Reduction and Fuel Oxidation Reactions

#### 4.1.1 Oxygen Reduction Reaction

The electroreduction of oxygen has two pathways, 2-electron and 4-electron reduction, leading to the formation of H_2_O_2_ and H_2_O, respectively.^80−82^ The 2-electron ORR pathway enables the electrosynthesis of H_2_O_2_, providing an energy-saving alternative to the conventional anthraquinone process.^83,84^ The 4-electron ORR pathway holds prominent importance as an electrochemical conversion process in metal–air batteries and proton exchange membrane fuel cells. Both pathways require highly efficient catalysts that can accelerate the reaction kinetics for activating O_2_ and breaking the O–O bond.^85−87^ Pt-based materials have been recognized as state-of-the-art catalysts, specifically following the 4-electron ORR pathway.^88,89^ However, the high cost and limited availability of Pt necessitate the urgent exploration of alternative catalysts that are cost-effective without sacrificing the ORR performance.

**Figure 5:**
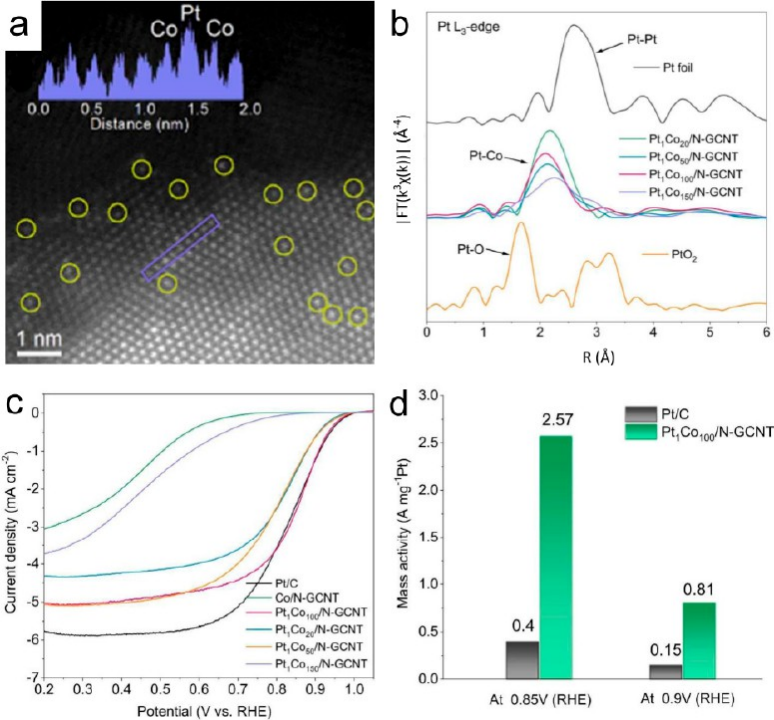
(a) HAADF-STEM image of Pt_1_Co_100_/N-GCNT. The insert in (a) displays the line-scan intensity profile obtained from the purple box. (b) Magnitude of the Fourier-transformed EXAFS spectra at the Pt L_3_-edge. (c) ORR polarization curves of the catalysts. (d) Mass activities of Pt/C (20 wt %) and Pt_1_Co_100_/N-GCNT at 0.85 and 0.9 V (vs RHE). Reproduced with permission from ref (90). Copyright 2022 Elsevier.

Incorporating Pt single atoms into a cost-effective metal host through doping enables the optimization of both the electronic and geometric structures of the catalysts. The SAA catalyst with Pt–Co dual sites encapsulated in N-doped graphitized carbon nanotubes (Pt_1_Co_*n*_/N-GCNT) consists of Pt single atoms dispersed on Co nanoparticles (Figure 5).^90^ The HAADF-STEM images indicate that Pt atoms were isolated on the surfaces of the Co nanoparticles. The authors stated that Pt–Co bonds formed in the Pt_1_Co_*n*_ SAA, but no Pt–Pt bonds were identified in the EXAFS spectra, suggesting the formation of Pt–Co SAAs. However, assigning the peak solely to the Pt–Co coordination bond based on peak positions in the EXAFS spectra might not be rigorously sufficient to prove the existence of Pt single atoms. ORR polarization curves show that the Pt_1_Co_100_/N-GCNT SAA catalyst achieved a mass activity of 0.81 A mg_Pt_^–1^ at 0.90 V vs RHE in 0.1 M HClO_4_ solution, superior to Co–N/GCNT and commercial Pt/C catalysts. The N-GCNT encapsulation protected SAA from corrosion in acidic environments. Density functional theory (DFT) calculations demonstrated that the Pt–Co dual sites in the SAA could promote the immobilization of *OOH and dissociation of *OH, which is beneficial to the 4-electron ORR pathway.

**Figure 6:**
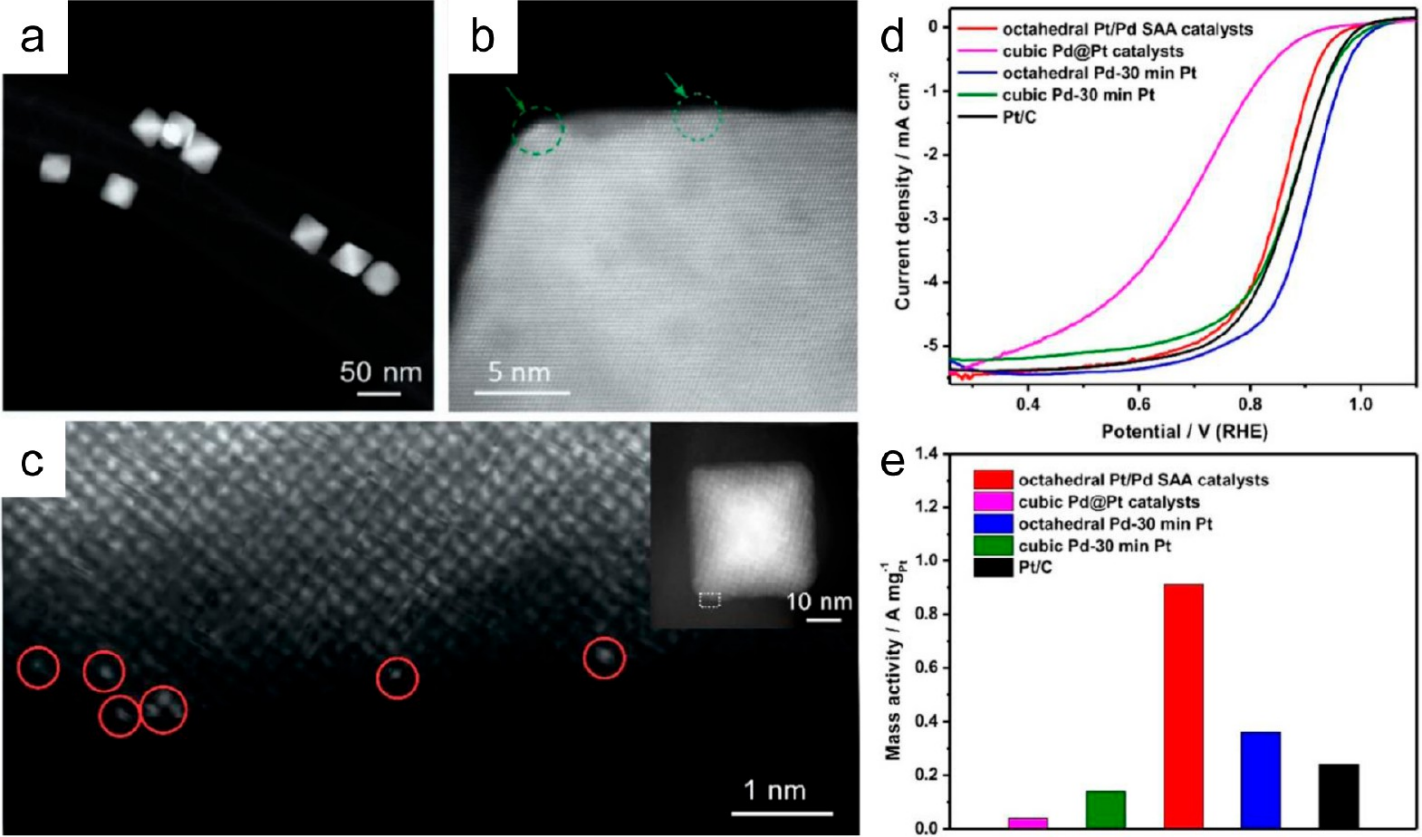
(a) Low-magnification STEM image of the octahedral Pt/Pd SAA catalysts on NCNTs. (b) Atomic-resolution STEM image of an individual octahedral Pt/Pd SAA particle. The green circled area indicates the presence of a few brighter atoms at the surface of the octahedral Pd particle. (c) High-resolution STEM image showing the surface of one individual octahedral Pt/Pd SAA (inset). The red circled area illustrates individual bright spots on the surface of octahedral Pd particles (d) ORR polarization curves of the catalysts. (e) Mass activities of the catalysts at 0.9 V_RHE_. Reproduced with permission from ref (91). Copyright 2019 American Chemical Society.

Similarly, Pt/Pd SAA catalysts on nitrogen-doped carbon nanotubes were synthesized by an ALD method (Figure 6).^91^ The STEM images demonstrated that single Pt atoms were dispersed on the octahedral Pd surfaces. The Pt/Pd SAA catalysts exhibited much higher ORR activities than the Pd@Pt core@shell catalysts and commercial Pt/C. According to the DFT calculations, the binding energy of *OH on Pt/Pd SAA was lower compared to that of Pd@Pt core@shell catalysts, leading to a mitigated strong adsorption of *OH and hence enhanced kinetics.

#### 4.1.2 Fuel Oxidation Reactions

In addition to proton exchange membrane fuel cells, small molecule oxidation reaction-based fuel cells, such as direct methanol fuel cells, direct ethanol fuel cells, and direct formic acid fuel cells, provide a further boost in energy density and ease of fuel transportation and storage. Nevertheless, achieving the complete oxidation of small molecules into CO_2_ and H_2_O poses significant challenges.^92−97^ Those small molecule oxidation reactions, including the methanol oxidation reaction (MOR), ethanol oxidation reaction (EOR), and formic acid oxidation reaction (FAOR), typically employ Pt-based alloy catalysts. The strong affinity of CO on the electrocatalysts commonly leads to the deactivation processes in small molecule oxidation-reaction-based fuel cells.^98,99^ In recent years, SAAs have received special attention as electrocatalysts for these reactions. One distinct feature of SAAs in these reactions is the potential for anti-CO poisoning.

Ru single atoms were deposited to the surface cavities of PtNi nanoparticles (Ru-ca-PtNi) via a selective ALD technique.^98^ The atomically dispersed Ru atoms were found to be specifically confined within the concave regime of PtNi. Ru-ca-PtNi demonstrated a record-high activity for MOR with a peak mass activity of 2.01 A mg_Pt_^–1^, which is a 5.8-fold enhancement over the commercial Pt/C catalyst. Operando electrochemical Fourier transform infrared spectroscopy (FTIR) and DFT calculations demonstrate that the atomically dispersed Ru atoms at the PtNi cavities facilitate the CO removal by the upshift of the d-band center. Furthermore, Ru single atoms situated at concave sites exhibited elevated diffusion barriers, thereby contributing to enhanced stability.

**Figure 7:**
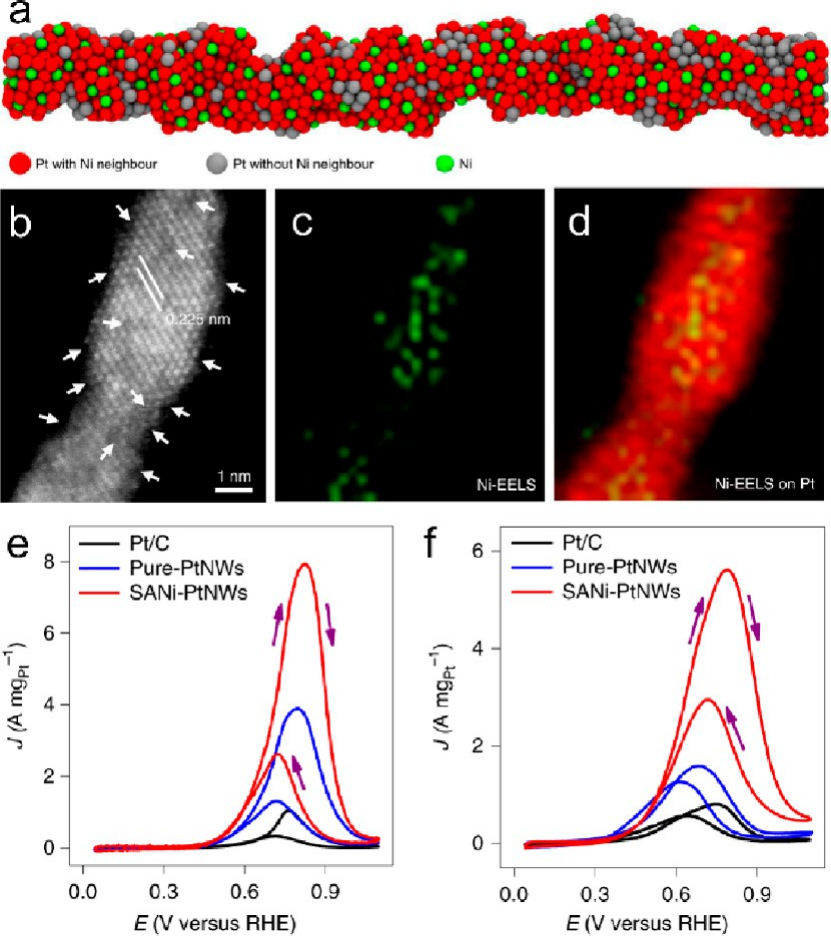
(a) Schematic diagram for SANi-PtNWs. (b) HAADF-STEM image of SANi-PtNWs. White arrows highlight the surface defects, steps, and concave cavity sites. (c) Ni EELS mapping. (d) Overlaid image of Ni-EELS mapping on Pt, with red representing Pt and green representing Ni. (e) MOR CV curves of the catalysts in a 1 M methanol + 1 M KOH solution. (F) EOR CV curves of the catalysts in a 1 M ethanol + 1 M KOH solution. Reproduced with permission from ref (100). Copyright 2019 Springer Nature.

A partial electrochemical dealloying method was developed to synthesize the NiPt SAA catalyst with atomically dispersed Ni atoms on Pt nanowires (SANi-PtNWs) (Figure 7).^100^ Isolated Ni atoms on the Pt surface contributed to the highly electrochemically active surface area. The SANi-PtNWs exhibited MOR and EOR activities in an alkaline medium with a mass activity of 7.93 ± 0.45 A mg_Pt_^–1^ and 5.60 ± 0.27 A mg_Pt_^–1^, respectively, much higher than those of PtNWs and commercial Pt/C catalyst.

The strain effect was investigated in SAA systems. For example, tensile-strained Pt-Rh SAA on an intermetallic PtBi nanoplate (PtBi@PtRh_1_),^101^ in which Rh was atomically dispersed in the tensile-strained Pt shell, exhibited better EOR performance than that of PtBi-Rh_1_ nanoplates, PtBi nanoplates, and commercial Pt/C catalysts. Benefiting from the isolated Rh single sites on a Pt shell with the tensile strain, the PtBi@PtRh_1_ SAA catalyst exhibited high selectivity toward the C_1_ pathway of complete oxidation. DFT calculations indicated that the synergy between Rh single atoms and tensile strain enhanced the adsorption of ethanol and its oxidative intermediates and promoted the cleavage of C–C bonds in the intermediates with a low activation energy. The engineering of strained SAA catalysts represents an appealing strategy for expediting the electrocatalyst development.

**Figure 8:**
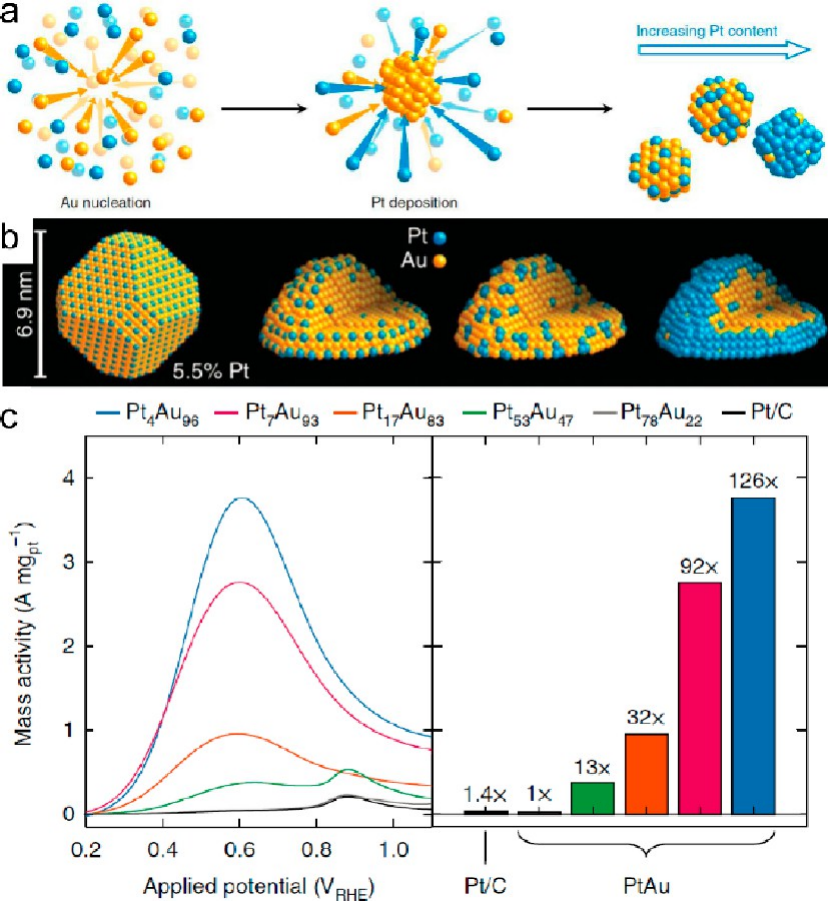
(a) Illustration of the nanoparticle formation via the reduction of solvated ions. (b) Schematic illustration for the PtAu alloy with different Pt coverage on the surface. (c) Pt mass-normalized anodic sweeps obtained from PtAu nanoparticle catalysts in 0.1 M HClO_4_ + 0.1 M HCOOH, with the peak currents graphed for comparison (left). Reproduced with permission from ref (102). Copyright 2018 Springer Nature.

SAA catalysts have also been investigated in direct formic acid fuel cells. A series of PtAu nanoparticles with a Pt composition ranging from 4% to 96% were synthesized by reducing Au and Pt chloride precursors (Figure 8).^102^ Structural analysis of Pt_4_Au_96_ and Pt_7_Au_93_ samples revealed a high surface density of low-coordinated Pt single atoms, which could prevent the catalysts from self-poisoning by CO on the surface. The anti-CO poison at the single atom Pt site led to superior FAOR catalytic activity and selectivity.

### 4.2 Water Splitting: Oxygen and Hydrogen Evolution Reactions

#### 4.2.1 Oxygen Evolution Reaction

**Figure 9:**
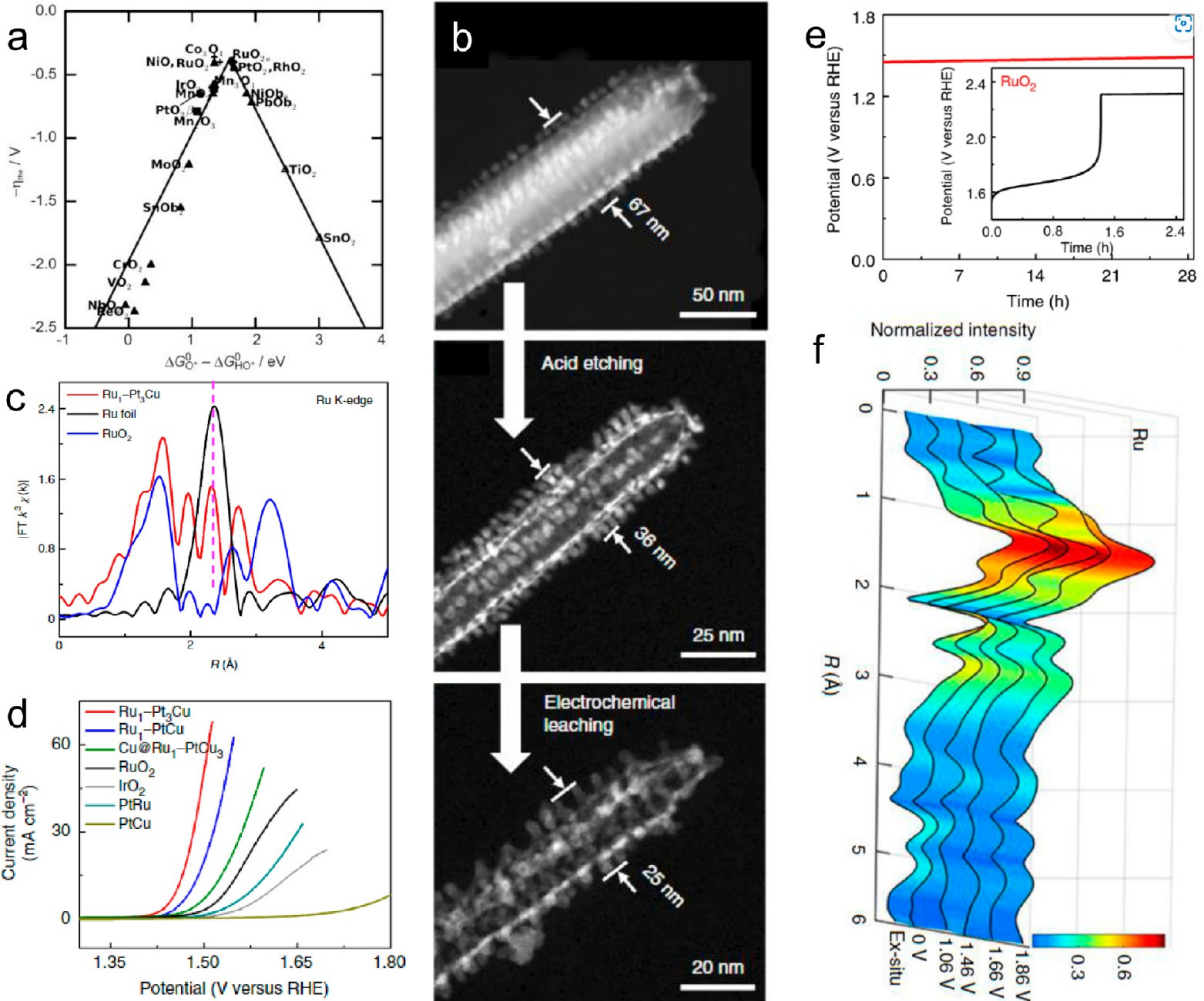
(a) Overpotential needed for the OER as a function of the difference between *O and *OH binding strength. RuO_2_ is shown at the top of the volcano map. Reproduced with permission from ref (108). Copyright 2011 Wiley. (b) HAADF-STEM images of three-dimensional visualization of tomographic reconstruction of Ru_1_-Pt_3_Cu. Reproduced with permission from ref (32). Copyright 2019 Springer Nature. (c) Ru K-edge EXAFS spectra of Ru_1_-Pt_3_Cu, Ru foil, and RuO_2_. (d) LSV curves of Ru_1_-Pt_3_Cu and reference samples for the acidic OER. (e) Stability test of Ru_1_-Pt_3_Cu through cyclic potential scanning and chronoamperometry. (f) In situ XAS spectra of the Ru K-edge of Ru_1_-Pt_3_Cu at various potentials. Reproduced with permission from ref (32). Copyright 2019 Springer Nature.

OER is a crucial anodic reaction in electrocatalytic water splitting. Efforts to develop efficient catalysts for acidic OER have intensified in recent years due to their implications in proton exchange membrane electrolyzers in industrial-scale applications.^103−105^ Ruthenium and its oxide are considered as the state-of-the-art OER catalysts in acidic conditions.^106,107^ The balance between the binding energy of two important intermediates, *O and *OH, moves RuO_2_ to the top of the volcano with the smallest overpotential (Figure 9a).^108^ Nevertheless, the long-term stability of RuO_2_ catalysts suffers from severe degradation in acidic solutions due to corrosion and subsequent leaching.

To enhance the OER stability, atomically dispersed Ru single atoms doped on PtCu alloys (Ru_1_-Pt_3_Cu) were prepared via sequential acid etching and electrochemical leaching (Figure 9b).^32^ Although the authors stated that there was no Ru–Ru bond observed in the Ru K-edge EXAFS spectrum, indicating the absence of Ru clusters (Figure 9c), relying only on peak positions in the EXAFS spectra might be insufficient for proving the existence of Ru single atoms. Ru_1_-Pt_3_Cu exhibits a superior OER performance in 0.1 M HClO_4_, with an enhanced activity and remarkable stability of 30 h at 10 mA/cm^2^, outperforming the state-of-the-art RuO_2_ catalyst (Figure 9d and 9e). In situ XAS analysis (Figure 9f) suggests the oxidation resistance of Ru, which was attributed to the charge compensation from Pt_3_Cu to Ru, mitigated the overoxidation of Ru.

#### 4.2.2 Hydrogen Evolution Reaction

**Figure 10:**
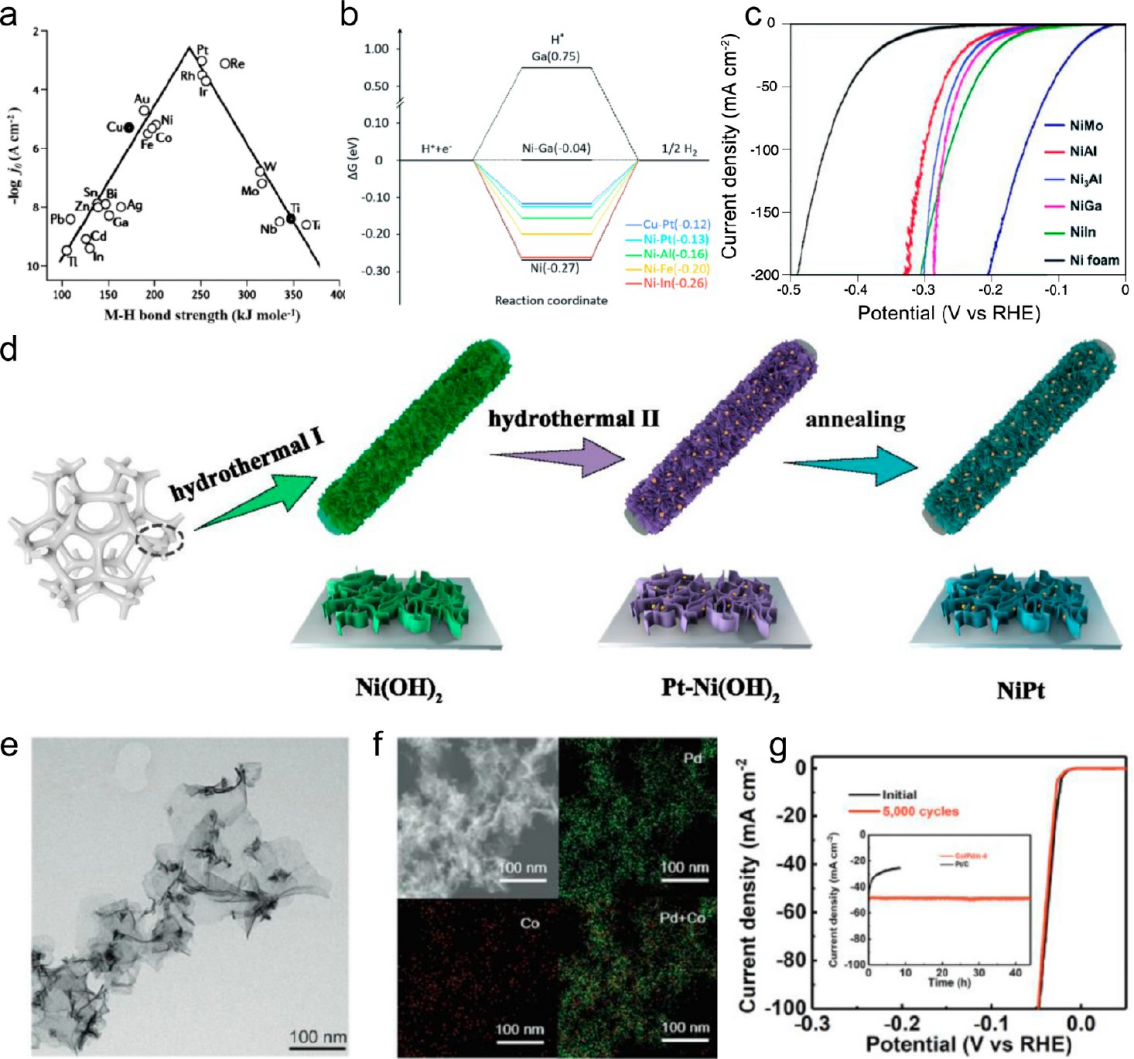
(a) Exchange currents for hydrogen evolution as a function of the metal–hydrogen binding strength. Reproduced with permission from ref (113). Copyright 1972 Elsevier. (b) Free energy diagrams for hydrogen evolution. Six candidates (NiGa, CuPt, NiPt, NiAl, NiFe, and NiIn) and two pure metals (Ni and Ga) are shown. (c) Typical LSV curves for five Ni-based candidates (NiMo, NiAl, Ni_3_Al, NiGa, and NiIn) compared with the pure Ni foam. Reproduced with permission from ref (115). Copyright 2021 Royal Society of Chemistry. (d) Synthetic process of NiPt single atom alloy on Ni foam. Reproduced with permission from ref (116). Copyright 2022 American Chemical Society. Characterizations of Co-Pdm-4: (e) TEM image and (f) HAADF-STEM image and corresponding EDS mappings. (g) Stability test of Co-Pdm-4 through cyclic potential scanning and chronoamperometric. Reproduced with permission from ref (117). Copyright 2023 Wiley.

HER is considered the most sustainable route to generate clean, renewable hydrogen.^109−111^ The binding energy for *H is a reliable descriptor for evaluating the catalyst HER activity.^112^ The *H binding energy for Pt is very close to the optimum value, leading to the highest activity of HER compared with other pure metals (Figure 10a).^113^ This is consistent with current experimental practice that Pt is the state-of-the-art metal for HER. Based on the *H binding energy, Levchenko et al. employed high-throughput computation and machine learning to identify more than 200 unreported SAA candidates that might show promising activity for HER.^114^ Wang et al. used an inverse catalyst design workflow which identified 70 binary and 752 ternary SAA candidates for HER.^115^ Corresponding experiments verified that homogeneously dispersed Ni-based bimetallic SAAs (NiMo, NiAl, Ni_3_Al, NiGa, and NiIn) showed improved catalytic performance compared with that of bare Ni foam (Figure 10b and 10c).

Pt single atoms were also incorporated into ultraporous Ni substrates (NiPt) by hydrothermal treatment followed by annealing (Figure 10d).^116^ The NiPt catalyst with only 0.3 wt % Pt loading exhibited excellent HER activity and durability in the alkaline solution. Non-Pt SAA catalysts have also been synthesized and investigated. Qin et al. reported homogeneously immobilized Co single atoms on the ultrathin two-dimensional Pd metallene (Co/Pdm-4) (Figures 10e and 10f).^117^ Co/Pdm exhibits remarkably enhanced HER activity and long-term stability compared to commercial Pt/C and Pd/C catalysts in acidic media (Figure 10g). Charge rearrangement was evidenced by the computational studies, indicating a reduced charge density around the Co single atoms. Au single atoms in Ru were fabricated via laser ablation in liquid.^118^ The RuAu SAA exhibited a low overpotential of 24 mV at 10 mA/cm^2^ for HER in an alkaline solution, comparable to commercial Pt/C catalysts. Nevertheless, it remains a grand challenge to develop nonprecious metal catalysts that can simultaneously achieve low overpotential, high activity, and stability for HER.

### 4.3 CO_2_ Reduction Reaction

Electrochemical CO_2_RR offers an alternative means to transform chemicals sustainably and store intermittent energy into chemical bonds.^7−10^ Theoretical studies have been performed on various SAAs to understand the activity and selectivity trend induced by dopants.^119,120^ Li et al. performed a theoretical study for transition metal (TM1 = Sc, Ti, V, Cr, Mn, Fe, Co, Ni, and Zn) single atom-doped Cu(111) for CO_2_RR.^121^ All systems were proposed to follow the pathway of *CO_2_ → *COOH → *CO. V/Cu(111) was found to demonstrate the lowest limiting potential due to the strong binding of *CO that promotes further reduction.

The presence of single-atom dopants in SAAs provides an asymmetric local environment, which could lead to the formation of multicarbon products. Ag single atoms in Cu were found to selectively reduce CO_2_ to multicarbon products and suppress the competing HER due to compressive strain and appropriate binding strength. Weak carbon binding of Ag destabilizes the C_2_H_4_ and promotes C_2_H_5_OH production.^122^ Ag atoms also induce the asymmetric compressive strain and ligand effect by altering the nearby Cu electronic structures, which have been shown to promote the C_1_–C_1_ and C_1_–C_2_ coupling in an Ag-doped Cu(111) model.^123^ A similar effect has been proposed by Sargent et al. that Ag-doped Cu SAA could selectively produce *n*-propanol in a CO reduction reaction.^124^

**Figure 11:**
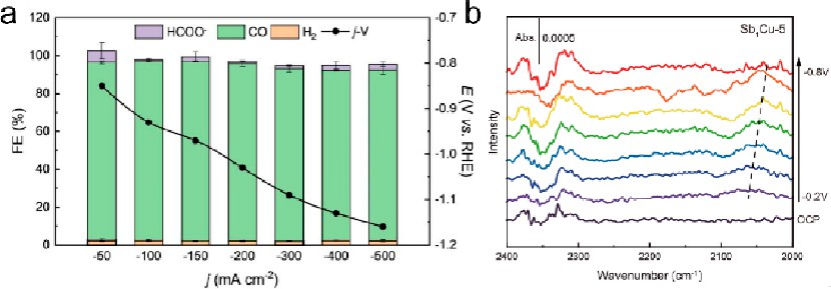
(a) FE and partial current density of Sb_1_Cu at various potentials. (b) In situ ATR-SEIRAS spectra of Sb_1_Cu-5. Reproduced with permission from ref (125). Copyright 2023 Springer Nature.

Single-atom dopants could also offer active sites that can selectively promote C_1_ production, such as CO and formic acid. An antimony–copper SAA (Sb_1_Cu) was reported to efficiently reduce CO_2_ to CO with a Faradaic efficiency (FE) higher than 95%, a lower onset potential, and a much suppressed C–C coupling compared to pure Cu (Figure 11a).^125^ In situ spectroscopic measurements and theoretical simulations reasoned that the atomic Sb–Cu interface in Cu promotes CO_2_ adsorption/activation and weakens the binding strength of *CO. In situ attenuated total reflection surface-enhanced infrared absorption spectroscopy (ATR-SEIRAS) spectra revealed a fingerprint infrared band at 2000–2100 cm^–1^, which was assigned to surface-bound CO (*CO) (Figure 11b). Additionally, the lower frequency of Sb_1_Cu-5 compared to Cu indicates weakened *CO adsorption and much lower CO coverage. Similarly, single-atom Pb-alloyed Cu catalyst (Pb_1_Cu) was reported to dominantly produce formic acid where CO_2_ was believed to be activated on the modulated Cu sites rather than the isolated Pb, shifting the reaction from the carboxyl to the formate pathway.^126^

**Figure 12:**
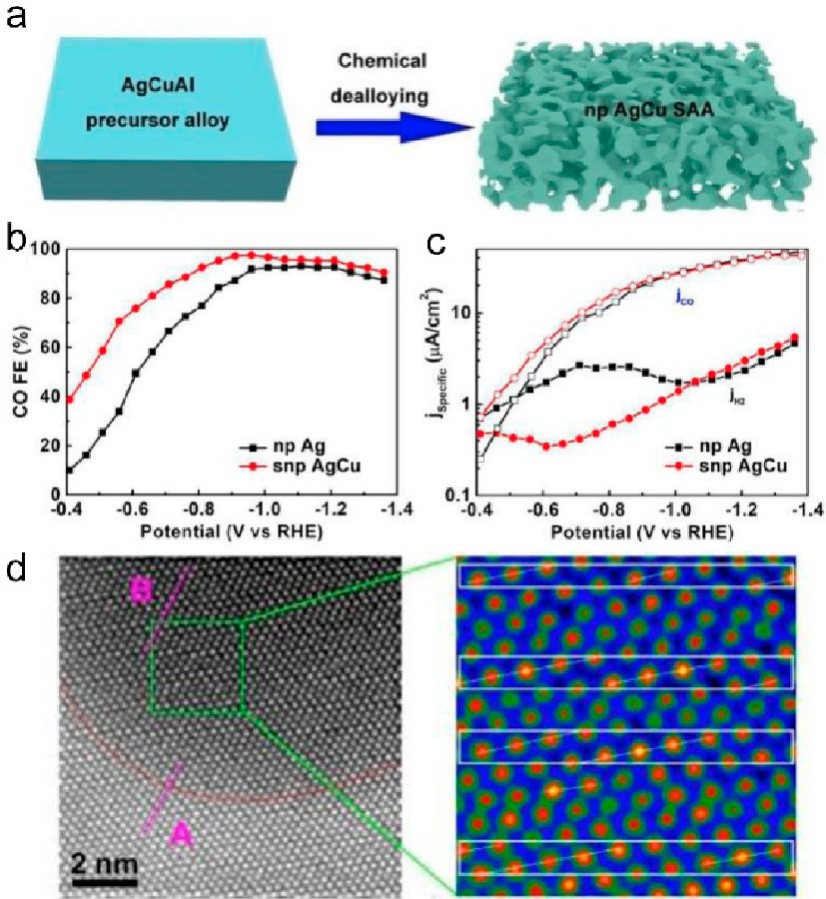
(a) Schematic illustration for synthesizing nanoporous (np) AgCu SAA via chemical dealloying. Electrocatalytic CO_2_ reduction reaction performance of snp AgCu. (b) FE of CO and (c) partial current density. (d) Atomically resolved HAADF image of snp AgCu and the magnification of the region framed in the green box of the Cu-rich domain. (red dots: Cu, brightened dots: Ag) Reproduced with permission from ref (127). Copyright 2022 Elsevier.

Lei et al. synthesized a nanoporous AgCu SAA (np AgCu SAA) with atomic ratio Cu:Ag = 1:110, which selectively reduced CO_2_ to CO.^127^ The catalyst was fabricated by the chemical dealloying process. Driven by the electrochemical CO_2_RR, a surface reconstruction took place (Figure 12a). The np AgCu SAA electrode after 3 h of electrolysis at −0.91 V was denoted as snp AgCu, which then achieved high selectivity and activity toward CO production (Figure 12b and 12c). The reconstructed catalyst configuration was investigated by the atomically resolved HAADF-STEM, and an evident interface between the Cu-rich region and Ag-rich region was observed (Figure 12d). The atomic structure at the edge of the ligament surface suggests that the Cu-rich domains reconstructed on the snp AgCu surface. The Cu-rich domain with a modulated structure on the surface led to a large Ag/Cu interfacial area.

### 4.4 Nitrate Reduction Reaction

The electrochemical NO_3_RR offers a promising alternative route to the energy-intensive Haber–Bosch process for NH_3_ production.^12,128^ NO_3_RR can employ the nitrate source from wastewater, which makes it a sustainable route for both wastewater treatment and ammonia production.^11,12^ Recently, SAAs have been studied as efficient electrocatalysts for the NO_3_RR.

**Figure 13:**
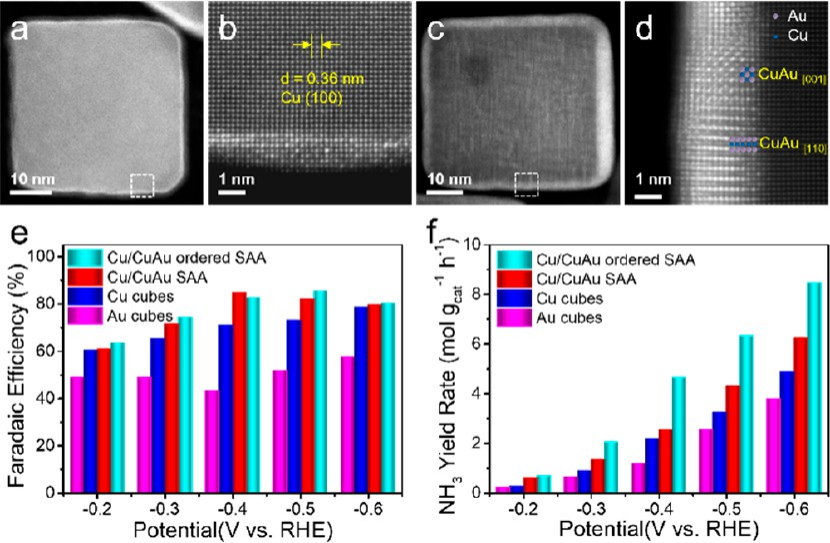
(a) HAADF-STEM and (b) atomic-resolution HAADF-STEM images of Cu/CuAu dilute SAA nanocubes. Figure b shows that dilute Au atoms are atomically dispersed on the surface of Cu nanocubes. (c) HAADF-STEM and (d) atomic-resolution HAADF-STEM images of Cu/CuAu ordered SAA nanocubes. Specifically, in part d, the CuAu shell shows an ordered body-centered tetragonal structure, in which Au atoms are isolated by Cu atoms. (e) FE of NH_3_ and (f) NH_3_ yield rates at different potentials for the catalysts. Reproduced with permission from ref (65). Copyright 2023 Springer Nature.

Our group reported a direct solution-phase synthesis of Cu/CuAu core/shell nanocubes with tunable SAA layers.^65^ Using Cu nanocubes as a template and through a seed-mediated colloidal synthesis, we controlled the density of single-sites and the number of atomic layers and obtained two types of SAAs: dilute Cu/CuAu SAA and ordered Cu/CuAu SAA (Figure 13). Both Cu/CuAu SAAs showed a higher NO_3_RR activity than Cu and Au nanocubes. Especially, the ordered Cu/CuAu SAA catalysts showed a high NH_3_ selectivity with a FE of 85.5% and an exceedingly high NH_3_ yield rate of 8.47 mol h^–1^ g^–1^. DFT calculations indicated that the high NO_3_RR activity of the Cu/CuAu SAA catalysts can be attributed to the (001)-oriented Cu site ensemble strengthening the *NO_3_ adsorption due to d-state hybridization of surface Cu atoms while weakening the *N binding due to strong Pauli repulsion from the subsurface Au single atoms.

**Figure 14:**
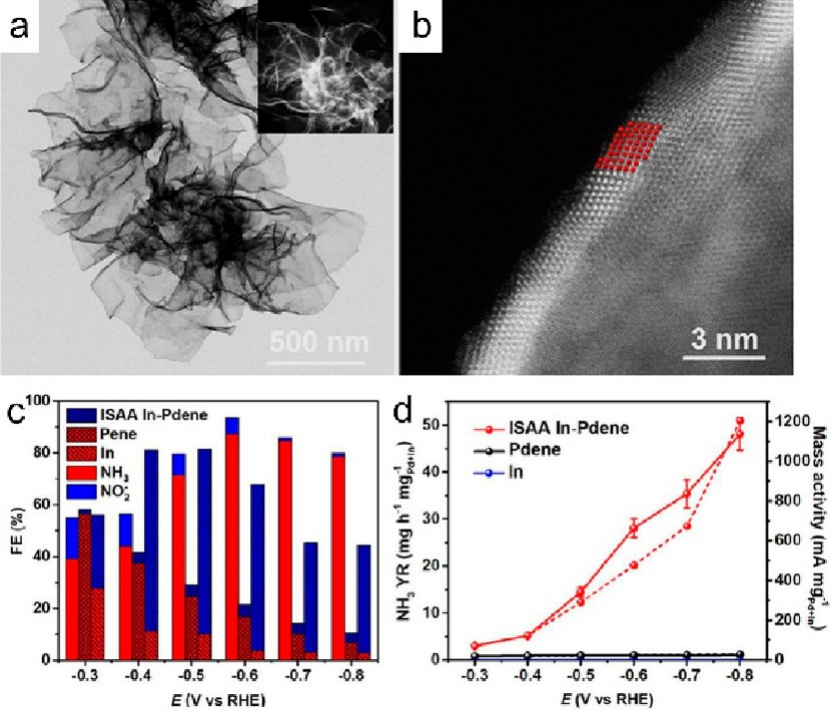
(a) TEM and (b) lateral HAADF-STEM images of ISAA In-Pdene with six atomic layers. (c) FE of different products and (d) the corresponding NH_3_ yield rate (solid lines) and mass activity (dashed lines) of ISAA In-Pdene and Pdene at different applied potentials. Reproduced with permission from ref (66). Copyright 2023 American Chemical Society.

Yu et al. reported an intermetallic SAA In–Pd bimetallene (ISAA In-Pdene) with six atomic layers, in which Pd single atoms are isolated by In atoms. This ISAA In-Pdene effectively catalyzed neutral NO_3_RR with an NH_3_ FE of 87.2% and an NH_3_ yield rate of 28.06 mg h^–1^ mg_Pd_^–1^ (Figure 14).^66^ Theoretical calculations revealed that the intermetallic SAA structure significantly reduces the overlap of Pd d orbitals and narrows the p–d hybridization of the Pd-d and In-p states near the Fermi level. This leads to enhanced nitrate adsorption and a lowered energy barrier for the potential-determining step of the NO_3_RR. They further designed a Zn-NO_3_^–^ flow battery with a Zn plate as the anode and the ISAA In-Pdene as the cathode, which delivered an FE of 93.4% for NH_3_ production and a high power density of 12.64 mW cm^–2^.

## 5 Challenges and Perspectives

The catalysis and materials community has witnessed remarkable advancements in the synthesis and characterization of SAA catalysts along with their applications in various crucial reactions with broad implications for energy conversion and chemical transformations. As such, this perspective summarizes the recent progress in the field of SAA nanoparticle catalysts for electrocatalysis. The discussion covers synthetic methods, the promoting effects of these SAA catalysts, and corresponding design strategies that could be transformative for future catalyst design in other reactions. Despite these achievements, as a relatively new structural motif gaining momentum, SAA catalysts still face many unaddressed challenges.

(1) Enhancing the catalytic performance of SAAs necessitates optimization to increase the number of single-atom active sites. SAAs commonly encounter challenges associated with low single-atom loading, as the sparsely distributed single atoms on the host metal’s surface can limit their activity. This limitation leaves ample room for improving catalytic performance through optimization of the density of single metals on the surface. However, a straightforward increase in atom dispersion may lead to aggregation, posing a considerable challenge in achieving high-density SAAs. Overcoming this dispersion limitation remains a significant challenge, prompting the exploration of innovative synthetic strategies. For instance, single-atom-based ordered intermetallic alloys have demonstrated superior electrocatalytic performance compared to their dilute SAA counterparts.^65,129^ Furthermore, there is a pressing need to intensify the development of synthesis methods for monodisperse SAA nanoparticles to provide a precise understanding of the structure–property relationships. Additionally, optimization of the SAAs is needed to further improve the catalytic performance while reducing the cost. The current design of SAAs mostly uses precious metals as the host metals, such as Pt and Pd. Using nonprecious metals for designing monometallic, bimetallic, or multimetallic hosts can offer potential opportunities to meet the need for low-cost SAAs and provide extra sites for adsorption of multidentate intermediates with tunable binding strength.
(2) Advanced electron microscopic techniques, such as HAADF-STEM, are indispensable tools for directly imaging single-atom sites on SAAs. However, the limited information on the specific local area in STEM images means that the insights gained may not fully represent the overall sample information. This limitation becomes particularly pronounced when dealing with significant sample heterogeneity. To comprehensively characterize SAAs, it is essential to integrate the XAS technique. XAS analysis provides detailed atomic information on both host and dopant metals, offering insights into bond information and coordination numbers from a unique perspective. This integration enhances the overall characterization of SAAs by providing a more thorough understanding of their structural and compositional aspects. We would like to emphasize, as elaborated in Section 3.2, it is crucial to underscore the importance of maintaining and ensuring adherence to rigorous data analysis standards in assigning the structures of SAAs, given the insensitivity of EXAFS to minority species. To substantiate claims, it is recommended to compare best fits with negative fits.
(3) More importantly, the potential dynamic structural evolution of SAAs calls for thorough investigation using high-resolution, time-resolved spectroscopic and microscopic probes. Given the high surface free energy of dopants at the surface layer, the structure may undergo reconstruction during prolonged electrochemical operation. Hence, in situ/operando characterizations, including high-resolution, time-resolved spectroscopic, and microscopic probes, play a pivotal role in attaining a thorough understanding of potential-driven chemical states and the dynamic evolution of atomic configurations in SAAs.
(4) Given the expansive design space, conducting experimental synthesis and characterization for all potential SAA candidates in reactions of interest is impractical. The emergence of theoretical calculations and machine learning has paved the way for a new design principle, offering screening and design guidance for novel and effective SAAs. Theoretical studies can provide valuable physical insights into catalyst design, and interpretable machine learning can then formulate a suitable framework to explore the vast design space of SAAs. This combined approach holds promise for efficiently identifying and optimizing SAAs for various applications. Furthermore, considering the reported synthetic approaches all require precise control over reaction parameters, addressing the scalability of SAA catalysts becomes imperative for their widespread applications. Perhaps, developing automated SAA synthesizers could herald a new era of future material and chemical manufacturing.

In addition to the representative electrocatalytic applications discussed above, SAAs have also found applications in the fields of thermocatalysis and photocatalysis. Recent advancements highlight the substantial potential of SAAs in emerging catalysis applications. For instance, Ye et al. designed the charge-polarized Pd^δ−^–Cu^δ+^ dual-site copper SAA toward efficient electrochemical C–N coupling for urea production.^130^ In summary, well-defined SAAs hold great promise for applications across diverse catalytic fields in the future. Realizing their full potential hinges on optimizing the synthesis, characterization, scalability, and fundamental understanding of SAAs. This necessitates sustained collaborative efforts from both the catalysis and materials communities.

## References

[ref1] Jiao K.; Xuan J.; Du Q.; Bao Z.; Xie B.; Wang B.; Zhao Y.; Fan L.; Wang H.; Hou Z.; Huo S.; Brandon N. P.; Yin Y.; Guiver M. D. Designing the Next Generation of Proton-Exchange Membrane Fuel Cells. Nature 2021, 595, 361 10.1038/s41586-021-03482-7. 34262215

[ref2] Zhong M.; Tran K.; Min Y.; Wang C.; Wang Z.; Dinh C.-T.; De Luna P.; Yu Z.; Rasouli A. S.; Brodersen P.; Sun S.; Voznyy O.; Tan C.-S.; Askerka M.; Che F.; Liu M.; Seifitokaldani A.; Pang Y.; Lo S.-C.; Ip A.; Ulissi Z.; Sargent E. H. Accelerated discovery of CO_2_ electrocatalysts using active machine learning. Nature 2020, 581 (7807), 178–183. 10.1038/s41586-020-2242-8. 32405017

[ref3] Roger I.; Shipman M. A.; Symes M. D. Earth-abundant catalysts for electrochemical and photoelectrochemical water splitting. Nat. Rev. Chem. 2017, 1 (1), 0003 10.1038/s41570-016-0003.

[ref4] Chung H. T.; Cullen D. A.; Higgins D.; Sneed B. T.; Holby E. F.; More K. L.; Zelenay P. Direct atomic-level insight into the active sites of a high-performance PGM-free ORR catalyst. Science 2017, 357 (6350), 479–484. 10.1126/science.aan2255. 28774924

[ref5] Laberty-Robert C.; Vallé K.; Pereira F.; Sanchez C. Design and properties of functional hybrid organic–inorganic membranes for fuel cells. Chem. Soc. Rev. 2011, 40 (2), 961–1005. 10.1039/c0cs00144a. 21218233

[ref6] Zhang J.; Zhang Q.; Feng X. Support and Interface Effects in Water-Splitting Electrocatalysts. Adv. Mater. 2019, 31 (31), 1808167 10.1002/adma.201808167. 30838688

[ref7] Zhao Y.; Hao L.; Ozden A.; Liu S.; Miao R. K.; Ou P.; Alkayyali T.; Zhang S.; Ning J.; Liang Y.; Xu Y.; Fan M.; Chen Y.; Huang J. E.; Xie K.; Zhang J.; O’Brien C. P.; Li F.; Sargent E. H.; Sinton D. Conversion of CO_2_ to multicarbon products in strong acid by controlling the catalyst microenvironment. Nat. Synth. 2023, 2 (5), 403–412. 10.1038/s44160-022-00234-x.

[ref8] Li F.; Thevenon A.; Rosas-Hernández A.; Wang Z.; Li Y.; Gabardo C. M.; Ozden A.; Dinh C. T.; Li J.; Wang Y.; Edwards J. P.; Xu Y.; McCallum C.; Tao L.; Liang Z.-Q.; Luo M.; Wang X.; Li H.; O’Brien C. P.; Tan C.-S.; Nam D.-H.; Quintero-Bermudez R.; Zhuang T.-T.; Li Y. C.; Han Z.; Britt R. D.; Sinton D.; Agapie T.; Peters J. C.; Sargent E. H. Molecular tuning of CO_2_-to-ethylene conversion. Nature 2020, 577 (7791), 509–513. 10.1038/s41586-019-1782-2. 31747679

[ref9] Ross M. B.; De Luna P.; Li Y.; Dinh C.-T.; Kim D.; Yang P.; Sargent E. H. Designing materials for electrochemical carbon dioxide recycling. Nat. Catal. 2019, 2 (8), 648–658. 10.1038/s41929-019-0306-7.

[ref10] Zhang W.; Hu Y.; Ma L.; Zhu G.; Wang Y.; Xue X.; Chen R.; Yang S.; Jin Z. Progress and Perspective of Electrocatalytic CO_2_ Reduction for Renewable Carbonaceous Fuels and Chemicals. Adv. Sci. 2018, 5 (1), 1700275 10.1002/advs.201700275. PMC5770696 29375961

[ref11] Wang Y.; Wang C.; Li M.; Yu Y.; Zhang B. Nitrate electroreduction: mechanism insight, in situ characterization, performance evaluation, and challenges. Chem. Soc. Rev. 2021, 50 (12), 6720–6733. 10.1039/D1CS00116G. 33969861

[ref12] Min B.; Gao Q.; Yan Z.; Han X.; Hosmer K.; Campbell A.; Zhu H. Powering the Remediation of the Nitrogen Cycle: Progress and Perspectives of Electrochemical Nitrate Reduction. Ind. Eng. Chem. Res. 2021, 60 (41), 14635–14650. 10.1021/acs.iecr.1c03072.

[ref13] Nitopi S.; Bertheussen E.; Scott S. B.; Liu X.; Engstfeld A. K.; Horch S.; Seger B.; Stephens I. E. L.; Chan K.; Hahn C.; Nørskov J. K.; Jaramillo T. F.; Chorkendorff I. Progress and Perspectives of Electrochemical CO_2_ Reduction on Copper in Aqueous Electrolyte. Chem. Rev. 2019, 119 (12), 7610–7672. 10.1021/acs.chemrev.8b00705. 31117420

[ref14] Seh Z. W.; Kibsgaard J.; Dickens C. F.; Chorkendorff I.; Nørskov J. K.; Jaramillo T. F. Combining theory and experiment in electrocatalysis: Insights into materials design. Science 2017, 355 (6321), aad4998 10.1126/science.aad4998. 28082532

[ref15] Zhu Y. P.; Guo C.; Zheng Y.; Qiao S.-Z. Surface and Interface Engineering of Noble-Metal-Free Electrocatalysts for Efficient Energy Conversion Processes. Acc. Chem. Res. 2017, 50 (4), 915–923. 10.1021/acs.accounts.6b00635. 28205437

[ref16] Ji S.; Chen Y.; Wang X.; Zhang Z.; Wang D.; Li Y. Chemical Synthesis of Single Atomic Site Catalysts. Chem. Rev. 2020, 120 (21), 11900–11955. 10.1021/acs.chemrev.9b00818. 32242408

[ref17] Wang A.; Li J.; Zhang T. Heterogeneous single-atom catalysis. Nat. Rev. Chem. 2018, 2 (6), 65–81. 10.1038/s41570-018-0010-1.

[ref18] Yang X.-F.; Wang A.; Qiao B.; Li J.; Liu J.; Zhang T. Single-Atom Catalysts: A New Frontier in Heterogeneous Catalysis. Acc. Chem. Res. 2013, 46 (8), 1740–1748. 10.1021/ar300361m. 23815772

[ref19] Liu J.-C.; Xiao H.; Li J. Constructing High-Loading Single-Atom/Cluster Catalysts via an Electrochemical Potential Window Strategy. J. Am. Chem. Soc. 2020, 142 (7), 3375–3383. 10.1021/jacs.9b06808. 31994381

[ref20] Chen Y.; Ji S.; Chen C.; Peng Q.; Wang D.; Li Y. Single-Atom Catalysts: Synthetic Strategies and Electrochemical Applications. Joule 2018, 2 (7), 1242–1264. 10.1016/j.joule.2018.06.019.

[ref21] Zhang T.; Walsh A. G.; Yu J.; Zhang P. Single-atom alloy catalysts: structural analysis, electronic properties and catalytic activities. Chem. Soc. Rev. 2021, 50 (1), 569–588. 10.1039/D0CS00844C. 33170202

[ref22] Hannagan R. T.; Giannakakis G.; Flytzani-Stephanopoulos M.; Sykes E. C. H. Single-Atom Alloy Catalysis. Chem. Rev. 2020, 120 (21), 12044–12088. 10.1021/acs.chemrev.0c00078. 32588624

[ref23] Greiner M. T.; Jones T. E.; Beeg S.; Zwiener L.; Scherzer M.; Girgsdies F.; Piccinin S.; Armbrüster M.; Knop-Gericke A.; Schlögl R. Free-atom-like d states in single-atom alloy catalysts. Nat. Chem. 2018, 10 (10), 1008–1015. 10.1038/s41557-018-0125-5. 30150725

[ref24] Kaiser S. K.; Chen Z.; Faust Akl D.; Mitchell S.; Pérez-Ramírez J. Single-Atom Catalysts across the Periodic Table. Chem. Rev. 2020, 120 (21), 11703–11809. 10.1021/acs.chemrev.0c00576. 33085890

[ref25] Da Y.; Jiang R.; Tian Z.; Han X.; Chen W.; Hu W. The applications of single-atom alloys in electrocatalysis: Progress and challenges. SmartMat 2023, 4 (1), e1136 10.1002/smm2.1136.

[ref26] Shen T.; Wang S.; Zhao T.; Hu Y.; Wang D. Recent Advances of Single-Atom-Alloy for Energy Electrocatalysis. Adv. Energy Mater. 2022, 12 (39), 2201823 10.1002/aenm.202201823.

[ref27] Wang Y.; Su H.; He Y.; Li L.; Zhu S.; Shen H.; Xie P.; Fu X.; Zhou G.; Feng C.; Zhao D.; Xiao F.; Zhu X.; Zeng Y.; Shao M.; Chen S.; Wu G.; Zeng J.; Wang C. Advanced Electrocatalysts with Single-Metal-Atom Active Sites. Chem. Rev. 2020, 120 (21), 12217–12314. 10.1021/acs.chemrev.0c00594. 33136387

[ref28] Jiang L.; Liu K.; Hung S.-F.; Zhou L.; Qin R.; Zhang Q.; Liu P.; Gu L.; Chen H. M.; Fu G.; Zheng N. Facet engineering accelerates spillover hydrogenation on highly diluted metal nanocatalysts. Nat. Nanotechnol. 2020, 15 (10), 848–853. 10.1038/s41565-020-0746-x. 32747741

[ref29] Kyriakou G.; Boucher M. B.; Jewell A. D.; Lewis E. A.; Lawton T. J.; Baber A. E.; Tierney H. L.; Flytzani-Stephanopoulos M.; Sykes E. C. H. Isolated Metal Atom Geometries as a Strategy for Selective Heterogeneous Hydrogenations. Science 2012, 335 (6073), 1209–1212. 10.1126/science.1215864. 22403387

[ref30] Spivey T. D.; Holewinski A. Selective Interactions between Free-Atom-like d-States in Single-Atom Alloy Catalysts and Near-Frontier Molecular Orbitals. J. Am. Chem. Soc. 2021, 143 (31), 11897–11902. 10.1021/jacs.1c04234. 34319717

[ref31] Yang T.; Qin F.; Zhang S.; Rong H.; Chen W.; Zhang J. Atomically dispersed Ru in Pt_3_Sn intermetallic alloy as an efficient methanol oxidation electrocatalyst. Chem. Commun. 2021, 57 (17), 2164–2167. 10.1039/D0CC08210D. 33524088

[ref32] Yao Y.; Hu S.; Chen W.; Huang Z.-Q.; Wei W.; Yao T.; Liu R.; Zang K.; Wang X.; Wu G.; Yuan W.; Yuan T.; Zhu B.; Liu W.; Li Z.; He D.; Xue Z.; Wang Y.; Zheng X.; Dong J.; Chang C.-R.; Chen Y.; Hong X.; Luo J.; Wei S.; Li W.-X.; Strasser P.; Wu Y.; Li Y. Engineering the electronic structure of single atom Ru sites via compressive strain boosts acidic water oxidation electrocatalysis. Nat. Catal. 2019, 2 (4), 304–313. 10.1038/s41929-019-0246-2.

[ref33] Lucci F. R.; Lawton T. J.; Pronschinske A.; Sykes E. C. H. Atomic Scale Surface Structure of Pt/Cu(111) Surface Alloys. J. Phys. Chem. C 2014, 118 (6), 3015–3022. 10.1021/jp405254z.

[ref34] Qi K.; Chhowalla M.; Voiry D. Single atom is not alone: Metal–support interactions in single-atom catalysis. Mater. Today 2020, 40, 173–192. 10.1016/j.mattod.2020.07.002.

[ref35] Qin R.; Liu P.; Fu G.; Zheng N. Strategies for Stabilizing Atomically Dispersed Metal Catalysts. Small Methods 2018, 2 (1), 1700286 10.1002/smtd.201700286.

[ref36] Darby M. T.; Sykes E. C. H.; Michaelides A.; Stamatakis M. Carbon Monoxide Poisoning Resistance and Structural Stability of Single Atom Alloys. Top. Catal. 2018, 61 (5), 428–438. 10.1007/s11244-017-0882-1. 31258304 PMC6560695

[ref37] Giannakakis G.; Flytzani-Stephanopoulos M.; Sykes E. C. H. Single-Atom Alloys as a Reductionist Approach to the Rational Design of Heterogeneous Catalysts. Acc. Chem. Res. 2019, 52 (1), 237–247. 10.1021/acs.accounts.8b00490. 30540456

[ref38] Yang C.; Miao Z.; Zhang F.; Li L.; Liu Y.; Wang A.; Zhang T. Hydrogenolysis of methyl glycolate to ethanol over a Pt–Cu/SiO_2_ single-atom alloy catalyst: a further step from cellulose to ethanol. Green Chem. 2018, 20 (9), 2142–2150. 10.1039/C8GC00309B.

[ref39] Pei G. X.; Liu X. Y.; Yang X.; Zhang L.; Wang A.; Li L.; Wang H.; Wang X.; Zhang T. Performance of Cu-Alloyed Pd Single-Atom Catalyst for Semihydrogenation of Acetylene under Simulated Front-End Conditions. Acs Catal. 2017, 7 (2), 1491–1500. 10.1021/acscatal.6b03293.

[ref40] Pei G. X.; Liu X. Y.; Wang A.; Lee A. F.; Isaacs M. A.; Li L.; Pan X.; Yang X.; Wang X.; Tai Z.; Wilson K.; Zhang T. Ag Alloyed Pd Single-Atom Catalysts for Efficient Selective Hydrogenation of Acetylene to Ethylene in Excess Ethylene. Acs Catal. 2015, 5 (6), 3717–3725. 10.1021/acscatal.5b00700.

[ref41] Aich P.; Wei H.; Basan B.; Kropf A. J.; Schweitzer N. M.; Marshall C. L.; Miller J. T.; Meyer R. Single-Atom Alloy Pd–Ag Catalyst for Selective Hydrogenation of Acrolein. J. Phys. Chem. C 2015, 119 (32), 18140–18148. 10.1021/acs.jpcc.5b01357.

[ref42] Liu R.; El Berch J. N.; House S.; Meil S. W.; Mpourmpakis G.; Porosoff M. D. Reactive Separations of CO/CO_2_ mixtures over Ru–Co Single Atom Alloys. Acs Catal. 2023, 13 (4), 2449–2461. 10.1021/acscatal.2c05110.

[ref43] Xing F.; Jeon J.; Toyao T.; Shimizu K.-i.; Furukawa S. A Cu–Pd single-atom alloy catalyst for highly efficient NO reduction. Chem. Sci. 2019, 10 (36), 8292–8298. 10.1039/C9SC03172C. 32110288 PMC7006621

[ref44] Sun G.; Zhao Z.-J.; Mu R.; Zha S.; Li L.; Chen S.; Zang K.; Luo J.; Li Z.; Purdy S. C.; Kropf A. J.; Miller J. T.; Zeng L.; Gong J. Breaking the scaling relationship via thermally stable Pt/Cu single atom alloys for catalytic dehydrogenation. Nat. Commun. 2018, 9 (1), 4454 10.1038/s41467-018-06967-8. 30367052 PMC6203812

[ref45] Sheng Y.; Liu Y.; Yin Y.; Zou X.; Ren J.; Wu B.; Wang X.; Lu X. Rh promotional effects on Pt–Rh alloy catalysts for chemoselective hydrogenation of nitrobenzene to p-aminophenol. Chem. Eng. J. 2023, 452, 139448 10.1016/j.cej.2022.139448.

[ref46] Xia X. H.; Wang Y.; Ruditskiy A.; Xia Y. N. 25th Anniversary Article: Galvanic Replacement: A Simple and Versatile Route to Hollow Nanostructures with Tunable and Well-Controlled Properties. Adv. Mater. 2013, 25 (44), 6313–6333. 10.1002/adma.201302820. 24027074

[ref47] Cheng H.; Wang C.; Qin D.; Xia Y. Galvanic Replacement Synthesis of Metal Nanostructures: Bridging the Gap between Chemical and Electrochemical Approaches. Acc. Chem. Res. 2023, 56 (7), 900–909. 10.1021/acs.accounts.3c00067. 36966410 PMC10077583

[ref48] Pattadar D. K.; Masitas R. A.; Stachurski C. D.; Cliffel D. E.; Zamborini F. P. Reversing the Thermodynamics of Galvanic Replacement Reactions by Decreasing the Size of Gold Nanoparticles. J. Am. Chem. Soc. 2020, 142 (45), 19268–19277. 10.1021/jacs.0c09426. 33140961

[ref49] Skrabalak S. E.; Chen J.; Sun Y.; Lu X.; Au L.; Cobley C. M.; Xia Y. Gold Nanocages: Synthesis, Properties, and Applications. Acc. Chem. Res. 2008, 41 (12), 1587–1595. 10.1021/ar800018v. 18570442 PMC2645935

[ref50] Hong X.; Wang D.; Cai S.; Rong H.; Li Y. Single-Crystalline Octahedral Au–Ag Nanoframes. J. Am. Chem. Soc. 2012, 134 (44), 18165–18168. 10.1021/ja3076132. 23088493

[ref51] Hong J. W.; Kang S. W.; Choi B.-S.; Kim D.; Lee S. B.; Han S. W. Controlled Synthesis of Pd–Pt Alloy Hollow Nanostructures with Enhanced Catalytic Activities for Oxygen Reduction. ACS Nano 2012, 6 (3), 2410–2419. 10.1021/nn2046828. 22360814

[ref52] Ji K.; Xu M.; Xu S.-M.; Wang Y.; Ge R.; Hu X.; Sun X.; Duan H. Electrocatalytic Hydrogenation of 5-Hydroxymethylfurfural Promoted by a Ru_1_Cu Single-Atom Alloy Catalyst. Angew. Chem., Int. Ed. 2022, 61 (37), e202209849 10.1002/anie.202209849. 35876073

[ref53] Liu W.; Feng H.; Yang Y.; Niu Y.; Wang L.; Yin P.; Hong S.; Zhang B.; Zhang X.; Wei M. Highly-efficient RuNi single-atom alloy catalysts toward chemoselective hydrogenation of nitroarenes. Nat. Commun. 2022, 13 (1), 3188 10.1038/s41467-022-30536-9. 35676245 PMC9178046

[ref54] Giannakakis G.; Trimpalis A.; Shan J.; Qi Z.; Cao S.; Liu J.; Ye J.; Biener J.; Flytzani-Stephanopoulos M. NiAu Single Atom Alloys for the Non-oxidative Dehydrogenation of Ethanol to Acetaldehyde and Hydrogen. Top. Catal. 2018, 61 (5), 475–486. 10.1007/s11244-017-0883-0.

[ref55] Ouyang M.; Papanikolaou K. G.; Boubnov A.; Hoffman A. S.; Giannakakis G.; Bare S. R.; Stamatakis M.; Flytzani-Stephanopoulos M.; Sykes E. C. H. Directing reaction pathways via in situ control of active site geometries in PdAu single-atom alloy catalysts. Nat. Commun. 2021, 12 (1), 1549 10.1038/s41467-021-21555-z. 33750788 PMC7943817

[ref56] Liu J.; Uhlman M. B.; Montemore M. M.; Trimpalis A.; Giannakakis G.; Shan J.; Cao S.; Hannagan R. T.; Sykes E. C. H.; Flytzani-Stephanopoulos M. Integrated Catalysis-Surface Science-Theory Approach to Understand Selectivity in the Hydrogenation of 1-Hexyne to 1-Hexene on PdAu Single-Atom Alloy Catalysts. Acs Catal. 2019, 9 (9), 8757–8765. 10.1021/acscatal.9b00491.

[ref57] Wrasman C. J.; Boubnov A.; Riscoe A. R.; Hoffman A. S.; Bare S. R.; Cargnello M. Synthesis of Colloidal Pd/Au Dilute Alloy Nanocrystals and Their Potential for Selective Catalytic Oxidations. J. Am. Chem. Soc. 2018, 140 (40), 12930–12939. 10.1021/jacs.8b07515. 30220200

[ref58] Lu J. Atomic Lego Catalysts Synthesized by Atomic Layer Deposition. Acc. Mater. Res. 2022, 3 (3), 358–368. 10.1021/accountsmr.1c00250.

[ref59] Wang H.; Luo Q.; Liu W.; Lin Y.; Guan Q.; Zheng X.; Pan H.; Zhu J.; Sun Z.; Wei S.; Yang J.; Lu J. Quasi Pd_1_Ni single-atom surface alloy catalyst enables hydrogenation of nitriles to secondary amines. Nat. Commun. 2019, 10 (1), 4998 10.1038/s41467-019-12993-x. 31676812 PMC6825208

[ref60] Zhang L.; Wang Q.; Li L.; Banis M. N.; Li J.; Adair K.; Sun Y.; Li R.; Zhao Z.-J.; Gu M.; Sun X. Single atom surface engineering: A new strategy to boost electrochemical activities of Pt catalysts. Nano Energy 2022, 93, 106813 10.1016/j.nanoen.2021.106813.

[ref61] Cao L.; Liu W.; Luo Q.; Yin R.; Wang B.; Weissenrieder J.; Soldemo M.; Yan H.; Lin Y.; Sun Z.; Ma C.; Zhang W.; Chen S.; Wang H.; Guan Q.; Yao T.; Wei S.; Yang J.; Lu J. Atomically dispersed iron hydroxide anchored on Pt for preferential oxidation of CO in H_2_. Nature 2019, 565 (7741), 631–635. 10.1038/s41586-018-0869-5. 30700869

[ref62] Lu J. A Perspective on New Opportunities in Atom-by-Atom Synthesis of Heterogeneous Catalysts Using Atomic Layer Deposition. Catal. Lett. 2021, 151 (6), 1535–1545. 10.1007/s10562-020-03412-8.

[ref63] Feng Q.; Zhao S.; Wang Y.; Dong J.; Chen W.; He D.; Wang D.; Yang J.; Zhu Y.; Zhu H.; Gu L.; Li Z.; Liu Y.; Yu R.; Li J.; Li Y. Isolated Single-Atom Pd Sites in Intermetallic Nanostructures: High Catalytic Selectivity for Semihydrogenation of Alkynes. J. Am. Chem. Soc. 2017, 139 (21), 7294–7301. 10.1021/jacs.7b01471. 28502173

[ref64] Li J.; Sun S. Intermetallic Nanoparticles: Synthetic Control and Their Enhanced Electrocatalysis. Acc. Chem. Res. 2019, 52 (7), 2015–2025. 10.1021/acs.accounts.9b00172. 31251036

[ref65] Gao Q.; Yao B.; Pillai H. S.; Zang W.; Han X.; Liu Y.; Yu S.-W.; Yan Z.; Min B.; Zhang S.; Zhou H.; Ma L.; Xin H.; He Q.; Zhu H. Synthesis of core/shell nanocrystals with ordered intermetallic single-atom alloy layers for nitrate electroreduction to ammonia. Nat. Synth. 2023, 2 (7), 624–634. 10.1038/s44160-023-00258-x.

[ref66] Xie M.; Tang S.; Li Z.; Wang M.; Jin Z.; Li P.; Zhan X.; Zhou H.; Yu G. Intermetallic Single-Atom Alloy In–Pd Bimetallene for Neutral Electrosynthesis of Ammonia from Nitrate. J. Am. Chem. Soc. 2023, 145 (25), 13957–13967. 10.1021/jacs.3c03432. 37335563

[ref67] Kübel C.; Voigt A.; Schoenmakers R.; Otten M.; Su D.; Lee T.-C.; Carlsson A.; Bradley J. Recent Advances in Electron Tomography: TEM and HAADF-STEM Tomography for Materials Science and Semiconductor Applications. Microsc. Microanal. 2005, 11 (5), 378–400. 10.1017/S1431927605050361. 17481320

[ref68] Muller D. A. Structure and bonding at the atomic scale by scanning transmission electron microscopy. Nat. Mater. 2009, 8 (4), 263–270. 10.1038/nmat2380. 19308085

[ref69] Muller D. A.; Kourkoutis L. F.; Murfitt M.; Song J. H.; Hwang H. Y.; Silcox J.; Dellby N.; Krivanek O. L. Atomic-Scale Chemical Imaging of Composition and Bonding by Aberration-Corrected Microscopy. Science 2008, 319 (5866), 1073–1076. 10.1126/science.1148820. 18292338

[ref70] Wen Y.; Ophus C.; Allen C. S.; Fang S.; Chen J.; Kaxiras E.; Kirkland A. I.; Warner J. H. Simultaneous Identification of Low and High Atomic Number Atoms in Monolayer 2D Materials Using 4D Scanning Transmission Electron Microscopy. Nano Lett. 2019, 19 (9), 6482–6491. 10.1021/acs.nanolett.9b02717. 31430158

[ref71] Luo S.; Zhang L.; Liao Y.; Li L.; Yang Q.; Wu X.; Wu X.; He D.; He C.; Chen W.; Wu Q.; Li M.; Hensen E. J. M.; Quan Z. A Tensile-Strained Pt-Rh Single-Atom Alloy Remarkably Boosts Ethanol Oxidation. Adv. Mater. 2021, 33 (17), e2008508 10.1002/adma.202008508. 33749954

[ref72] Varela M.; Lupini A. R.; Benthem K. v.; Borisevich A. Y.; Chisholm M. F.; Shibata N.; Abe E.; Pennycook S. J. MATERIALS CHARACTERIZATION IN THE ABERRATION-CORRECTED SCANNING TRANSMISSION ELECTRON MICROSCOPE. Annu. Rev. Mater. Res. 2005, 35 (1), 539–569. 10.1146/annurev.matsci.35.102103.090513.

[ref73] Jiang N. Electron beam damage in oxides: a review. Rep. Prog. Phys. 2016, 79 (1), 016501 10.1088/0034-4885/79/1/016501. 26684361

[ref74] Ercius P.; Alaidi O.; Rames M. J.; Ren G. Electron Tomography: A Three-Dimensional Analytic Tool for Hard and Soft Materials Research. Adv. Mater. 2015, 27 (38), 5638–5663. 10.1002/adma.201501015. 26087941 PMC4710474

[ref75] Gurman S. J. EXAFS studies in materials science. J. Mater. Sci. 1982, 17 (6), 1541–1570. 10.1007/BF00540779.

[ref76] Frenkel A. I. Applications of extended X-ray absorption fine-structure spectroscopy to studies of bimetallic nanoparticle catalysts. Chem. Soc. Rev. 2012, 41 (24), 8163–78. 10.1039/c2cs35174a. 22833100

[ref77] Newton M. A.; Dent A. J.; Evans J. Bringing time resolution to EXAFS: recent developments and application to chemical systems. Chem. Soc. Rev. 2002, 31 (2), 83–95. 10.1039/b100115a. 12109208

[ref78] Li X.; Shen P.; Luo Y.; Li Y.; Guo Y.; Zhang H.; Chu K. PdFe Single-Atom Alloy Metallene for N_2_ Electroreduction. Angew. Chem., Int. Ed. 2022, 61 (28), e202205923 10.1002/anie.202205923. 35522475

[ref79] Alba-Rubio A.; Schweitzer N.; Gounder R.; Rioux R.; Bhan A.; Flaherty D.; Stangland E.; Bond J.; Schaidle J.; Notestein J.; Stowers K.; Welch L.; Personick M.; Chu P.; Christopher P.; Bollini P.; Meyer R., Addressing Rigor and Reproducibility in Thermal, Heterogeneous Catalysis; Zenodo, 2023. 10.5281/zenodo.8029159.

[ref80] Jiang Y.; Ni P.; Chen C.; Lu Y.; Yang P.; Kong B.; Fisher A.; Wang X. Selective Electrochemical H_2_O_2_ Production through Two-Electron Oxygen Electrochemistry. Adv. Energy Mater. 2018, 8 (31), 1801909 10.1002/aenm.201801909.

[ref81] Siahrostami S.; Verdaguer-Casadevall A.; Karamad M.; Deiana D.; Malacrida P.; Wickman B.; Escudero-Escribano M.; Paoli E. A.; Frydendal R.; Hansen T. W.; Chorkendorff I.; Stephens I. E. L.; Rossmeisl J. Enabling direct H_2_O_2_ production through rational electrocatalyst design. Nat. Mater. 2013, 12 (12), 1137–1143. 10.1038/nmat3795. 24240242

[ref82] Wang Y.; Waterhouse G. I. N.; Shang L.; Zhang T. Electrocatalytic Oxygen Reduction to Hydrogen Peroxide: From Homogenous to Heterogenous Electrocatalysis. Adv. Energy Mater. 2021, 11 (15), 2003323 10.1002/aenm.202003323.

[ref83] Xia C.; Xia Y.; Zhu P.; Fan L.; Wang H. Direct electrosynthesis of pure aqueous H_2_O_2_ solutions up to 20% by weight using a solid electrolyte. Science 2019, 366 (6462), 226–231. 10.1126/science.aay1844. 31601767

[ref84] Jirkovský J. S.; Panas I.; Ahlberg E.; Halasa M.; Romani S.; Schiffrin D. J. Single Atom Hot-Spots at Au–Pd Nanoalloys for Electrocatalytic H_2_O_2_ Production. J. Am. Chem. Soc. 2011, 133 (48), 19432–19441. 10.1021/ja206477z. 22023652

[ref85] An L.; Wei C.; Lu M.; Liu H.; Chen Y.; Scherer G. G.; Fisher A. C.; Xi P.; Xu Z. J.; Yan C. H. Recent Development of Oxygen Evolution Electrocatalysts in Acidic Environment. Adv. Mater. 2021, 33, 2006328 10.1002/adma.202006328. 33768614

[ref86] Adabi H.; Shakouri A.; Ul Hassan N.; Varcoe J. R.; Zulevi B.; Serov A.; Regalbuto J. R.; Mustain W. E. High-performing commercial Fe–N–C cathode electrocatalyst for anion-exchange membrane fuel cells. Nature Energy 2021, 6 (8), 834–843. 10.1038/s41560-021-00878-7.

[ref87] Luo F.; Roy A.; Silvioli L.; Cullen D. A.; Zitolo A.; Sougrati M. T.; Oguz I. C.; Mineva T.; Teschner D.; Wagner S.; Wen J.; Dionigi F.; Kramm U. I.; Rossmeisl J.; Jaouen F.; Strasser P. P-block single-metal-site tin/nitrogen-doped carbon fuel cell cathode catalyst for oxygen reduction reaction. Nat. Mater. 2020, 19 (11), 1215–1223. 10.1038/s41563-020-0717-5. 32661387

[ref88] Zhu H.; Zhang S.; Su D.; Jiang G.; Sun S. Surface Profile Control of FeNiPt/Pt Core/Shell Nanowires for Oxygen Reduction Reaction. Small 2015, 11 (29), 3545–3549. 10.1002/smll.201500330. 25786658

[ref89] Zhu H.; Zhang S.; Guo S.; Su D.; Sun S. Synthetic Control of FePtM Nanorods (M = Cu, Ni) To Enhance the Oxygen Reduction Reaction. J. Am. Chem. Soc. 2013, 135 (19), 7130–7133. 10.1021/ja403041g. 23634823

[ref90] Cheng X.; Wang Y.; Lu Y.; Zheng L.; Sun S.; Li H.; Chen G.; Zhang J. Single-atom alloy with Pt-Co dual sites as an efficient electrocatalyst for oxygen reduction reaction. Appl. Catal. B: Environ. 2022, 306, 121112 10.1016/j.apcatb.2022.121112.

[ref91] Zhang L.; Liu H.; Liu S.; Norouzi Banis M.; Song Z.; Li J.; Yang L.; Markiewicz M.; Zhao Y.; Li R.; Zheng M.; Ye S.; Zhao Z.-J.; Botton G. A.; Sun X. Pt/Pd Single-Atom Alloys as Highly Active Electrochemical Catalysts and the Origin of Enhanced Activity. Acs Catal. 2019, 9 (10), 9350–9358. 10.1021/acscatal.9b01677.

[ref92] Zhang Z.; Liu J.; Wang J.; Wang Q.; Wang Y.; Wang K.; Wang Z.; Gu M.; Tang Z.; Lim J.; Zhao T.; Ciucci F. Single-atom catalyst for high-performance methanol oxidation. Nat. Commun. 2021, 12 (1), 5235 10.1038/s41467-021-25562-y. 34475400 PMC8413426

[ref93] Yang X.; Wang Q.; Qing S.; Gao Z.; Tong X.; Yang N. Modulating Electronic Structure of an Au-Nanorod-Core-PdPt-Alloy-Shell Catalyst for Efficient Alcohol Electro-Oxidation. Adv. Energy Mater. 2021, 11 (26), 2100812 10.1002/aenm.202100812.

[ref94] Qiu Y.; Zhang J.; Jin J.; Sun J.; Tang H.; Chen Q.; Zhang Z.; Sun W.; Meng G.; Xu Q.; Zhu Y.; Han A.; Gu L.; Wang D.; Li Y. Construction of Pd-Zn dual sites to enhance the performance for ethanol electro-oxidation reaction. Nat. Commun. 2021, 12 (1), 5273 10.1038/s41467-021-25600-9. 34489455 PMC8421426

[ref95] Yin P.-F.; Zhou M.; Chen J.; Tan C.; Liu G.; Ma Q.; Yun Q.; Zhang X.; Cheng H.; Lu Q.; Chen B.; Chen Y.; Zhang Z.; Huang J.; Hu D.; Wang J.; Liu Q.; Luo Z.; Liu Z.; Ge Y.; Wu X.-J.; Du X.-W.; Zhang H. Synthesis of Palladium-Based Crystalline@Amorphous Core–Shell Nanoplates for Highly Efficient Ethanol Oxidation. Adv. Mater. 2020, 32 (21), 2000482 10.1002/adma.202000482. 32253801

[ref96] Li Z.; Chen Y.; Ji S.; Tang Y.; Chen W.; Li A.; Zhao J.; Xiong Y.; Wu Y.; Gong Y.; Yao T.; Liu W.; Zheng L.; Dong J.; Wang Y.; Zhuang Z.; Xing W.; He C.-T.; Peng C.; Cheong W.-C.; Li Q.; Zhang M.; Chen Z.; Fu N.; Gao X.; Zhu W.; Wan J.; Zhang J.; Gu L.; Wei S.; Hu P.; Luo J.; Li J.; Chen C.; Peng Q.; Duan X.; Huang Y.; Chen X.-M.; Wang D.; Li Y. Iridium single-atom catalyst on nitrogen-doped carbon for formic acid oxidation synthesized using a general host–guest strategy. Nat. Chem. 2020, 12 (8), 764–772. 10.1038/s41557-020-0473-9. 32541950

[ref97] Zhang W.; Yang Y.; Huang B.; Lv F.; Wang K.; Li N.; Luo M.; Chao Y.; Li Y.; Sun Y.; Xu Z.; Qin Y.; Yang W.; Zhou J.; Du Y.; Su D.; Guo S. Ultrathin PtNiM (M = Rh, Os, and Ir) Nanowires as Efficient Fuel Oxidation Electrocatalytic Materials. Adv. Mater. 2019, 31 (15), 1805833 10.1002/adma.201805833. 30803065

[ref98] Kong F.; Liu X.; Song Y.; Qian Z.; Li J.; Zhang L.; Yin G.; Wang J.; Su D.; Sun X. Selectively Coupling Ru Single Atoms to PtNi Concavities for High-Performance Methanol Oxidation via d-Band Center Regulation. Angew. Chem., Int. Ed. 2022, 61 (42), e202207524 10.1002/anie.202207524. 36038511

[ref99] Liu J.; Lucci F. R.; Yang M.; Lee S.; Marcinkowski M. D.; Therrien A. J.; Williams C. T.; Sykes E. C. H.; Flytzani-Stephanopoulos M. Tackling CO Poisoning with Single-Atom Alloy Catalysts. J. Am. Chem. Soc. 2016, 138 (20), 6396–6399. 10.1021/jacs.6b03339. 27167705

[ref100] Li M.; Duanmu K.; Wan C.; Cheng T.; Zhang L.; Dai S.; Chen W.; Zhao Z.; Li P.; Fei H.; Zhu Y.; Yu R.; Luo J.; Zang K.; Lin Z.; Ding M.; Huang J.; Sun H.; Guo J.; Pan X.; Goddard W. A.; Sautet P.; Huang Y.; Duan X. Single-atom tailoring of platinum nanocatalysts for high-performance multifunctional electrocatalysis. Nat. Catal. 2019, 2 (6), 495–503. 10.1038/s41929-019-0279-6.

[ref101] Luo S.; Zhang L.; Liao Y.; Li L.; Yang Q.; Wu X.; Wu X.; He D.; He C.; Chen W.; Wu Q.; Li M.; Hensen E. J. M.; Quan Z. A Tensile-Strained Pt–Rh Single-Atom Alloy Remarkably Boosts Ethanol Oxidation. Adv. Mater. 2021, 33 (17), 2008508 10.1002/adma.202008508. 33749954

[ref102] Duchesne P. N.; Li Z. Y.; Deming C. P.; Fung V.; Zhao X.; Yuan J.; Regier T.; Aldalbahi A.; Almarhoon Z.; Chen S.; Jiang D.-e.; Zheng N.; Zhang P. Golden single-atomic-site platinum electrocatalysts. Nat. Mater. 2018, 17 (11), 1033–1039. 10.1038/s41563-018-0167-5. 30250176

[ref103] Lei Z.; Cai W.; Rao Y.; Wang K.; Jiang Y.; Liu Y.; Jin X.; Li J.; Lv Z.; Jiao S.; Zhang W.; Yan P.; Zhang S.; Cao R. Coordination modulation of iridium single-atom catalyst maximizing water oxidation activity. Nat. Commun. 2022, 13 (1), 24 10.1038/s41467-021-27664-z. 35013202 PMC8748886

[ref104] Kuai C.; Xu Z.; Xi C.; Hu A.; Yang Z.; Zhang Y.; Sun C.-J.; Li L.; Sokaras D.; Dong C.; Qiao S.-Z.; Du X.-W.; Lin F. Phase segregation reversibility in mixed-metal hydroxide water oxidation catalysts. Nat. Catal. 2020, 3 (9), 743–753. 10.1038/s41929-020-0496-z.

[ref105] Kim J. S.; Kim B.; Kim H.; Kang K. Recent Progress on Multimetal Oxide Catalysts for the Oxygen Evolution Reaction. Adv. Energy Mater. 2018, 8 (11), 1702774 10.1002/aenm.201702774.

[ref106] Wu Z.-Y.; Chen F.-Y.; Li B.; Yu S.-W.; Finfrock Y. Z.; Meira D. M.; Yan Q.-Q.; Zhu P.; Chen M.-X.; Song T.-W.; Yin Z.; Liang H.-W.; Zhang S.; Wang G.; Wang H. Non-iridium-based electrocatalyst for durable acidic oxygen evolution reaction in proton exchange membrane water electrolysis. Nat. Mater. 2023, 22 (1), 100–108. 10.1038/s41563-022-01380-5. 36266572

[ref107] Chen F.-Y.; Wu Z.-Y.; Adler Z.; Wang H. Stability challenges of electrocatalytic oxygen evolution reaction: From mechanistic understanding to reactor design. Joule 2021, 5 (7), 1704–1731. 10.1016/j.joule.2021.05.005.

[ref108] Man I. C.; Su H.-Y.; Calle-Vallejo F.; Hansen H. A.; Martínez J. I.; Inoglu N. G.; Kitchin J.; Jaramillo T. F.; Nørskov J. K.; Rossmeisl J. Universality in Oxygen Evolution Electrocatalysis on Oxide Surfaces. ChemCatChem. 2011, 3 (7), 1159–1165. 10.1002/cctc.201000397.

[ref109] Li Z.; Niu W.; Yang Z.; Kara A.; Wang Q.; Wang M.; Gu M.; Feng Z.; Du Y.; Yang Y. Boosting alkaline hydrogen evolution: the dominating role of interior modification in surface electrocatalysis. Energy Environ. Sci. 2020, 13 (9), 3110–3118. 10.1039/D0EE01750G.

[ref110] Fang S.; Zhu X.; Liu X.; Gu J.; Liu W.; Wang D.; Zhang W.; Lin Y.; Lu J.; Wei S.; Li Y.; Yao T. Uncovering near-free platinum single-atom dynamics during electrochemical hydrogen evolution reaction. Nat. Commun. 2020, 11 (1), 1029 10.1038/s41467-020-14848-2. 32098951 PMC7042219

[ref111] Tian X.; Zhao P.; Sheng W. Hydrogen Evolution and Oxidation: Mechanistic Studies and Material Advances. Adv. Mater. 2019, 31 (31), 1808066 10.1002/adma.201808066. 30932265

[ref112] Zheng Y.; Jiao Y.; Vasileff A.; Qiao S.-Z. The Hydrogen Evolution Reaction in Alkaline Solution: From Theory, Single Crystal Models, to Practical Electrocatalysts. Angew. Chem., Int. Ed. 2018, 57 (26), 7568–7579. 10.1002/anie.201710556. 29194903

[ref113] Trasatti S. Work function, electronegativity, and electrochemical behaviour of metals: III. Electrolytic hydrogen evolution in acid solutions. J. electroanal. chem. interfacial electrochem. 1972, 39 (1), 163–184. 10.1016/S0022-0728(72)80485-6.

[ref114] Han Z.-K.; Sarker D.; Ouyang R.; Mazheika A.; Gao Y.; Levchenko S. V. Single-atom alloy catalysts designed by first-principles calculations and artificial intelligence. Nat. Commun. 2021, 12 (1), 1833 10.1038/s41467-021-22048-9. 33758170 PMC7988173

[ref115] Zhou C.; Zhao J. Y.; Liu P. F.; Chen J.; Dai S.; Yang H. G.; Hu P.; Wang H. Towards the object-oriented design of active hydrogen evolution catalysts on single-atom alloys. Chem. Sci. 2021, 12 (31), 10634–10642. 10.1039/D1SC01018B. 34447556 PMC8356813

[ref116] Huo L.; Jin C.; Tang J.; Xu X.; Jiang K.; Shang L.; Li Y.; Zhang J.; Zhu L.; Chu J.; Hu Z. Ultrathin NiPt Single-Atom Alloy for Synergistically Accelerating Alkaline Hydrogen Evolution. ACS Appl. Energy Mater. 2022, 5 (12), 15136–15145. 10.1021/acsaem.2c02793.

[ref117] Yang S.; Si Z.; Li G.; Zhan P.; Liu C.; Lu L.; Han B.; Xie H.; Qin P. Single Cobalt Atoms Immobilized on Palladium-Based Nanosheets as 2D Single-Atom Alloy for Efficient Hydrogen Evolution Reaction. Small 2023, 19 (15), 2207651 10.1002/smll.202207651. 36631281

[ref118] Chen C.-H.; Wu D.; Li Z.; Zhang R.; Kuai C.-G.; Zhao X.-R.; Dong C.-K.; Qiao S.-Z.; Liu H.; Du X.-W. Ruthenium-Based Single-Atom Alloy with High Electrocatalytic Activity for Hydrogen Evolution. Adv. Energy Mater. 2019, 9 (20), 1803913 10.1002/aenm.201803913.

[ref119] Li M.; Hua B.; Wang L.-C.; Zhou Z.; Stowers K. J.; Ding D. Discovery of single-atom alloy catalysts for CO_2_-to-methanol reaction by density functional theory calculations. Catal. Today 2022, 388–389, 403–409. 10.1016/j.cattod.2020.04.059.

[ref120] Wang D.; Cao R.; Hao S.; Liang C.; Chen G.; Chen P.; Li Y.; Zou X. Accelerated prediction of Cu-based single-atom alloy catalysts for CO_2_ reduction by machine learning. Green Energy Environ. 2023, 8 (3), 820–830. 10.1016/j.gee.2021.10.003.

[ref121] Jiang J.-C.; Chen J.-C.; Zhao M.-d.; Yu Q.; Wang Y.-G.; Li J. Rational design of copper-based single-atom alloy catalysts for electrochemical CO_2_ reduction. Nano Res. 2022, 15 (8), 7116–7123. 10.1007/s12274-022-4476-2.

[ref122] Li Y. C.; Wang Z.; Yuan T.; Nam D.-H.; Luo M.; Wicks J.; Chen B.; Li J.; Li F.; de Arquer F. P. G.; Wang Y.; Dinh C.-T.; Voznyy O.; Sinton D.; Sargent E. H. Binding Site Diversity Promotes CO_2_ Electroreduction to Ethanol. J. Am. Chem. Soc. 2019, 141 (21), 8584–8591. 10.1021/jacs.9b02945. 31067857

[ref123] Clark E. L.; Hahn C.; Jaramillo T. F.; Bell A. T. Electrochemical CO_2_ Reduction over Compressively Strained CuAg Surface Alloys with Enhanced Multi-Carbon Oxygenate Selectivity. J. Am. Chem. Soc. 2017, 139 (44), 15848–15857. 10.1021/jacs.7b08607. 28988474

[ref124] Wang X.; Wang Z.; Zhuang T.-T.; Dinh C.-T.; Li J.; Nam D.-H.; Li F.; Huang C.-W.; Tan C.-S.; Chen Z.; Chi M.; Gabardo C. M.; Seifitokaldani A.; Todorović P.; Proppe A.; Pang Y.; Kirmani A. R.; Wang Y.; Ip A. H.; Richter L. J.; Scheffel B.; Xu A.; Lo S.-C.; Kelley S. O.; Sinton D.; Sargent E. H. Efficient upgrading of CO to C_3_ fuel using asymmetric C-C coupling active sites. Nat. Commun. 2019, 10 (1), 5186 10.1038/s41467-019-13190-6. 31780655 PMC6882816

[ref125] Li J.; Zeng H.; Dong X.; Ding Y.; Hu S.; Zhang R.; Dai Y.; Cui P.; Xiao Z.; Zhao D.; Zhou L.; Zheng T.; Xiao J.; Zeng J.; Xia C. Selective CO_2_ electrolysis to CO using isolated antimony alloyed copper. Nat. Commun. 2023, 14 (1), 340 10.1038/s41467-023-35960-z. 36670129 PMC9860050

[ref126] Zheng T.; Liu C.; Guo C.; Zhang M.; Li X.; Jiang Q.; Xue W.; Li H.; Li A.; Pao C.-W.; Xiao J.; Xia C.; Zeng J. Copper-catalysed exclusive CO_2_ to pure formic acid conversion via single-atom alloying. Nat. Nanotechnol. 2021, 16 (12), 1386–1393. 10.1038/s41565-021-00974-5. 34531557

[ref127] Wang H.; Zhou X.; Yu T.; Lu X.; Qian L.; Liu P.; Lei P. Surface restructuring in AgCu single-atom alloy catalyst and self-enhanced selectivity toward CO_2_ reduction. Electrochim. Acta 2022, 426, 140774 10.1016/j.electacta.2022.140774.

[ref128] Li P.; Liao L.; Fang Z.; Su G.; Jin Z.; Yu G. A multifunctional copper single-atom electrocatalyst aerogel for smart sensing and producing ammonia from nitrate. Proc. Natl. Acad. Sci. U.S.A. 2023, 120 (26), e2305489120 10.1073/pnas.2305489120. 37339226 PMC10293845

[ref129] Gao Q.; Pillai H. S.; Huang Y.; Liu S.; Mu Q.; Han X.; Yan Z.; Zhou H.; He Q.; Xin H.; Zhu H. Breaking adsorption-energy scaling limitations of electrocatalytic nitrate reduction on intermetallic CuPd nanocubes by machine-learned insights. Nat. Commun. 2022, 13 (1), 2338 10.1038/s41467-022-29926-w. 35487883 PMC9054787

[ref130] Xu M.; Wu F.; Zhang Y.; Yao Y.; Zhu G.; Li X.; Chen L.; Jia G.; Wu X.; Huang Y.; Gao P.; Ye W. Kinetically matched C–N coupling toward efficient urea electrosynthesis enabled on copper single-atom alloy. Nat. Commun. 2023, 14 (1), 6994 10.1038/s41467-023-42794-2. 37914723 PMC10620222

